# Absolute quantification of eight human milk oligosaccharides in breast milk to evaluate their concentration profiles and associations with infants’ neurodevelopmental outcomes

**DOI:** 10.1111/1750-3841.17597

**Published:** 2024-12-10

**Authors:** Keigo Sato, Yoshitaka Nakamura, Kazuhito Fujiyama, Kinuko Ohneda, Takahiro Nobukuni, Soichi Ogishima, Satoshi Mizuno, Seizo Koshiba, Shinichi Kuriyama, Shinji Jinno

**Affiliations:** ^1^ Food Microbiology and Function Research Laboratory Meiji Co., Ltd. Hachioji Japan; ^2^ Wellness Science Labs Meiji Holdings Co., Ltd. Hachioji Japan; ^3^ International Centre for Biotechnology Osaka University Suita Japan; ^4^ Tohoku Medical Megabank Organization Tohoku University Sendai Japan; ^5^ Graduate School of Medicine Tohoku University Sendai Japan; ^6^ Advanced Research Center for Innovations in Next‐Generation Medicine Tohoku University Sendai Japan; ^7^ International Research Institute of Disaster Science Tohoku University Sendai Japan

**Keywords:** breast milk, human milk oligosaccharides, head growth, infants, neurodevelopment

## Abstract

Human milk oligosaccharides (HMOs) have been positively associated with child neurodevelopment in some cohort studies. However, there is a lack of consistency in the association between HMOs and benefits to infants’ brains. Moreover, the quantification methods for HMOs have not yet been standardized. In this study, we developed a quantification method for evaluating eight HMOs (2′‐fucosyllactose [2′‐FL], 3′‐fucosyllactose [3′‐FL], 3′‐sialyllactose [3′‐SL], 6′‐sialyllactose [6′‐SL], lactosialyltetrasaccharide a [LSTa], lactosialyltetrasaccharide b [LSTb], lactosialyltetrasaccharide c [LSTc], and disialyllacto‐N‐tetraose [DSLNT]) in breast milk. After validating the method, we applied it to 1‐month breast milk samples (*n* = 150) from the Tohoku Medical Megabank Project Birth and Three‐Generation Cohort Study to assess HMO profiles in breast milk and their possible association with changes in head circumference z‐score (ΔHCZ) and neurodevelopmental scores of children (as measured by the Ages and Stages Questionnaire, Third Edition). The validation demonstrated that the method had relative standard deviation ≤ 12.7% of precision and 79.5–110.9% of accuracy. Using this method, eight HMO levels (2′‐FL, 0–4.74 mg/mL; 3′‐FL, 0.02–1.52 mg/mL; 3′‐SL, 0.07–0.32 mg/mL; 6′‐SL, 0.01–0.70 mg/mL; LSTa, 0.002–0.043 mg/mL; LSTb, 0.02–0.31 mg/mL; LSTc, 0.001–0.47 mg/mL; and DSLNT, 0.09–0.71 mg/mL [min–max, all participants]) and the ratio of low secretors (16.0%) in the Japanese cohort were obtained. The obtained HMO levels in breast milk were subjected to multivariate analysis to screen for HMOs showing a positive association with ΔHCZ and neurodevelopmental scores. The results proposed that ΔHCZ was positively associated with LSTb and 2′‐FL levels, whereas neurodevelopmental scores were positively associated with 2′‐FL levels (among all participants) and 3′‐SL and DSLNT levels (among secretor participants). This study showed that the developed method provides HMO profiles in Japanese breast milk, as well as additional information on the associations between specific HMOs and neurodevelopment, reinforcing the sum of evidence for the role of HMOs in the brain.

## 1 INTRODUCTION

Breast milk is a food that contains all the nutrients necessary for the growth of infants in early life. The World Health Organization (WHO) recommends exclusive breastfeeding until 6 months of age (World Health Organization, 2001). If breastfeeding is not possible, infant formula serves as a breast milk substitute. To develop a highly complete breast milk substitute or support good breastfeeding practices, several studies investigated the nutritional roles of various breast milk components (Christian et al., 2021; Dror & Allen, 2018).

Human milk oligosaccharides (HMOs) are one of the major components of breast milk that have been studied for their nutritional function and have been developed for products for infants by several researchers and manufacturers. HMOs are the third most abundant component of breast milk after lactose and lipids (Newburg & Neubauer, 1995), reaching the large intestine without digestion and serving as important prebiotic nutrients (Brand‐Miller et al., 1998). Lactose forms the core of HMO structures and branches into > 150 different structures through the association of *N*‐acetylglucosamine, galactose, fucose, and *N*‐acetylneuraminic acid (sialic acid) (Tadasu Urashima et al., 2018). The HMO profile in breast milk is considerably affected by four genotypes, which are identified by the status of secretor (*Sec*) (+)/(–) and Lewis (*Le*) (+)/(–) (Zhang et al., 2021). Genes associated with the HMO profiles are the α1,2‐fucosyltransferase (*FUT2*) gene for *Sec* status and the α1,3/4‐fucosyltransferase (*FUT3*) gene for *Le* status. The largest variation in breast milk HMOs lies in 2′‐fucosyllactose (2′‐FL), affected by *Sec* status. Breast milk 2′‐FL is the most abundant HMO in *Sec* (+) breast milk (Thurl et al., 2017), whereas the breast milk of *Sec* (–) mothers has markedly lower 2′‐FL levels. Infant containing major HMOs, such as 2′‐FL, 3′‐sialyllactose (3′‐SL), and 6′‐sialyllactose (6′‐SL), are now available on the market.

Although a positive association between the breast milk HMO levels and infants’ neurodevelopmental outcomes has been reported recently (Berger et al., 2020; Cho et al., 2021; Oliveros et al., 2020; Willemsen et al., 2023), further studies are necessary to better understand the exact impact of each HMO on specific neurodevelopmental aspects. Among HMOs, sialylated HMOs are expected to play an important role in the neurodevelopment of infants by supplying sialic acids to brains. Sialic acids are enriched in brain tissues and nervous systems (Schnaar et al., 2014) and are more abundant in breast‐fed infants than in formula‐fed infants, in the form of gangliosides and glycoproteins (Wang et al., 2003). Additionally, it is reported that sialylated HMOs affect cognitive scores in animals (Hauser et al., 2021; Obelitz‐Ryom et al., 2019; Oliveros et al., 2018; Pisa et al., 2023). In cohort studies, neurodevelopmental scores of children were positively associated with breast milk 6′‐SL levels (Oliveros et al., 2020) and 3′‐SL levels (in the A tetra + group) (Cho et al., 2021). The most abundant HMO in *Sec* (+), 2′‐FL, is also reported to positively associated with neurodevelopment in some animal studies (Oliveros et al., 2016; Vazquez et al., 2016) and cohort studies (Berger et al., 2020; Oliveros et al., 2020; Willemsen et al., 2023). A cohort by Mansell et al. (2023) reported that breast milk 2′‐FL is associated with head growth, one of the outcomes closely related to neurodevelopment (Cheong et al., 2008; Zhu et al., 2022). Although the benefits of breast milk HMOs on neurodevelopment in infants are becoming clearer, not all cohort studies have detected this association (Jorgensen et al., 2021; Menzel et al., 2021; Samuel et al., 2022; Sprenger et al., 2017). Moreover, there are few intervention studies examining the effects of HMOs on children's brain development. Further studies on the association between HMO levels in breast milk and infants’ neurodevelopmental scores or head growth are needed to better understand the nutritional role of HMOs in infants.

Accurate and reliable quantification methods can reveal the association between HMO levels in breast milk and infants’ neurodevelopment. Various methods have been developed to measure the HMO levels in breast milk. The high‐performance liquid chromatography (HPLC) method, utilizing fluorescent labeling, can quantify HMOs even in the absence of commercially available standards (Asakuma et al., 2007, 2008; Austin & Bénet, 2018). The liquid chromatography‐mass spectrometry (LC‐MS) method, which monitors precursor ions, requires no labeling procedures (Tonon et al., 2019). The multiple reaction monitoring (MRM) method of LC‐MS/MS, which monitors product ions, has higher selectivity than LC‐MS (Bao et al., 2013; Zhang et al., 2019). Additionally, higher sensitivity has been obtained with recent improvements in the performance of LC‐MS/MS instruments. Measurement methods should be chosen according to the instrument available in each laboratory. Additionally, most measurement methods require analytical standards, which present limited commercial availability. Therefore, these limitations complicate the establishment of standard measurement methods and require the development of accurate and reliable techniques based on the available equipment and analytical standards in each laboratory.

In this study, we aimed to develop absolute quantification methods for particular HMOs to evaluate the clinical samples of breast milk using the LC‐MS/MS approach by combining LC separation using a porous graphite carbon (PGC) column and a high‐resolution multiple reaction monitoring (MRM^HR^). The benefits of these instruments have been previously reported; the PGC column provides a good separation for isomer glycans (Ruhaak et al., 2009), and the MRM^HR^ provides several benefits, including its ease of use, sensitivity, and flexibility in selecting transitions post‐acquisition (Heil et al., 2021). We selected eight HMOs (2′‐FL, 3′‐FL, 3′‐SL, 6′‐SL, lactosialyltetrasaccharide a [LSTa], lactosialyltetrasaccharide b [LSTb], lactosialyltetrasaccharide c [LSTc], and disialyllacto‐*N*‐tetraose [DSLNT]), which are possibly related to neurodevelopment, and their isomer HMOs, as target analytes: (i) sialylated HMOs, whose analytical standards were commercially available (3′‐SL, 6′‐SL, LSTa, LSTb, LSTc, and DSLNT), and (ii) 2′‐FL and its isomer, 3′‐FL. After method validation, the quantification methods were used to evaluate the breast milk from the Tohoku Medical Megabank Project Birth and Three‐Generation Cohort Study (TMM BirThree cohort). Here, we conducted the following investigations: (i) an assessment of HMO profiles in Japanese breast milk, and (ii) the first exploratory study in Japan to evaluate associations between HMO levels in breast milk and head growth (change in head circumference [HC] z‐score [ΔHCZ]) or neurodevelopmental scores (Age and Stages Questionnaire, Third Edition [ASQ‐3]). In the latter study, a subgroup analysis among *Sec* (+) participants was performed to eliminate major genetic factors in HMOs secretion, since the *Sec* genotype influences the 2′‐FL levels, representing the most drastic variation among all HMO levels in breast milk. However, Pérez‐Escalante et al. (2022) reported that *Sec* (–) mothers typically account for only 15–20% of the population. Therefore, we did not perform a detailed subgroup analysis among *Sec* (–) participants due to the small number of individuals available to evaluate their associations with infant outcomes.

## 2 MATERIALS AND METHODS

### 2.1 HMO standards

**FIGURE 1:**
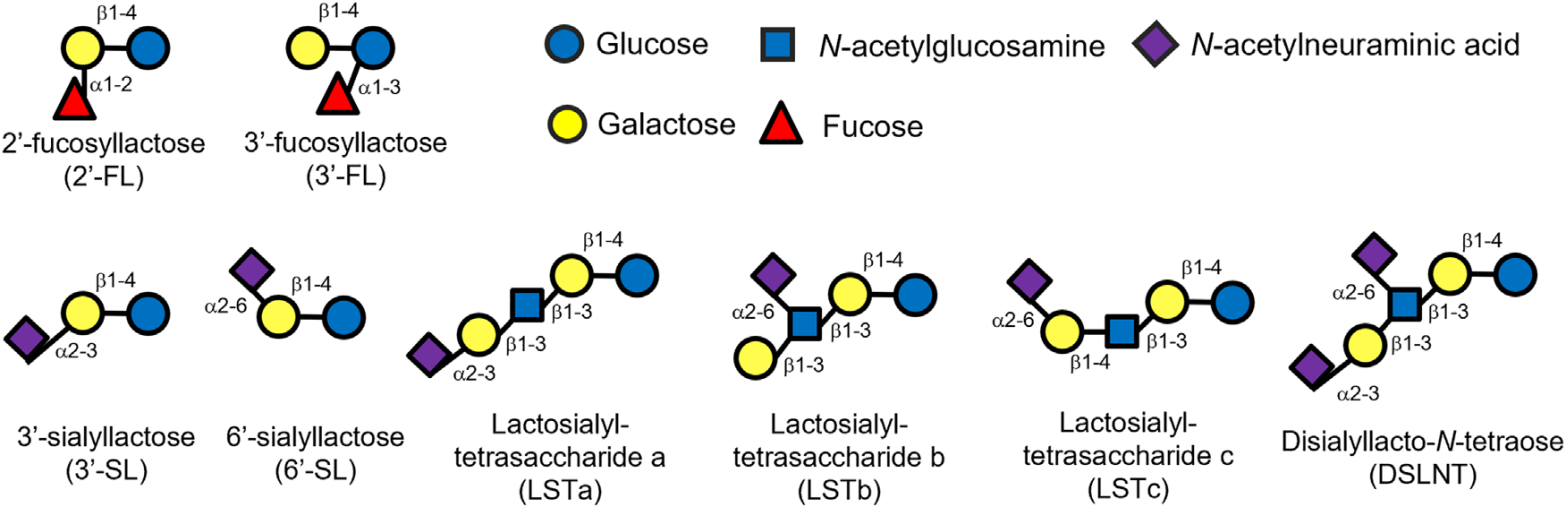
Structures of the eight human milk oligosaccharides quantified in this study.

Analytical standards of 2′‐FL, 3′‐FL, LSTa, LSTb, LSTc, and DSLNT were purchased from Biosynth. Analytical standards of 3′‐SL and 6′‐SL were purchased from Tokyo Chemical Industry. Figure 1 shows the structure of HMOs measured in this study.

### 2.2 Samples

The standard breast milk (SRM1953) was purchased from the National Institute of Standards and Technology and used for method validation. The information on SRM1953 is reported in a study by Schantz et al. (2013). Bovine milk was obtained from our factory and used for the spike and recovery test.

### 2.3 Sample preparation

Milk samples were thawed in room temperature and sonicated in a 40°C water bath for 10 min. Thereafter, 50 µL of aliquot was centrifuged for 15 min at 4°C and 4000 × *g*. After centrifugation, 10 µL of aliquot was mixed with 20 µL of ethanol. After 1 h at 4°C, the fraction was centrifuged for 10 min at 4°C and 4000 × *g*. The supernatant was mixed with 30 µL of 0.1 M sodium tetrahydroborate and incubated for 30 min at 65°C. After incubation, 30 µL of 0.1 M acetic acid was added. After centrifugation of the mixture for 5 min at 4°C and 12,000 × *g*, the supernatant was used as a test sample.

### 2.4 HMO analysis using the LC‐MS system

Chromatographic analysis was performed using a Supel Carbon LC column (2.1 × 100 mm; particle size, 2.7 µm) from Merck coupled with the LC‐MS/MS system (LC, Exion AC; MS/MS, TripleTOF 6600+, AB SCIEX). The analysis was separately performed for FL (2′‐FL and 3′‐FL) and SL (3′‐SL, 6′‐SL, LSTa, LSTb, LSTc, and DSLNT) measurements.

For FL measurements, the flow rate was 0.25 mL/min. The column was maintained at 40°C. A gradient mobile phase comprising 0.1% (v/v) formic acid in water (solvent A) and 0.1% (v/v) formic acid in acetonitrile (solvent B) was used. The gradient conditions were as follows: 0–2 min, 5 to 5% B; 2–10 min, 5 to 25% B; 10–12.5 min, 100 to 100% B; and 12.5–15 min, 5 to 5% B. The operating parameters were set as follows: positive‐ion mode; Ion Source Gas 1 (GS1), 10; Ion Source Gas 2 (GS2), 20; Curtain Gas (CUR), 15; Temperature (TEM), 550°C; ionSpray Voltage Floating (ISVF), 4500 V; declustering potential (DP), 80 V; Ion Release Delay (IRD), 66; and Ion Release Width (IRW), 24.

For sialylated HMO measurements, the flow rate was 0.6 mL/min. The column was maintained at 80°C. A gradient mobile phase comprising 0.1% (v/v) formic acid and 0.1% (v/v) diethylamine (DEA) in water (solvent C) and 0.1% (v/v) formic acid, 0.2% DEA, and 25% acetonitrile in isopropanol (solvent D) was used. The gradient conditions were as follows: 0–2 min, 0 to 0% D; 2–2.5 min, 0 to 10% D; 2.5–10 min, 10 to 12.5% D; 10–15 min, 12.5 to 60% D; 15–17.5 min, 80 to 80%; and 17.5–20 min, 0 to 0%. The operating parameters were set as follows: negative‐ion mode; GS1, 32; GS2, 35; CUR, 31 Pa; TEM, 550°C; ISVF, –4500 V; DP, –80 V; IRD, 66; IRW, 24.

In each measurement, the MS and MS/MS systems were operated using an MRM^HR^ (Table 1). The quantification standard curves were prepared with external standards of each HMO, with concentrations of 0.15625, 0.3125, 0.625, 1.25, 2.5, 5, 10, and 20 pmol/µL, with 1/x weighting. The test samples were quantified in the appropriate dilution by interpolating within the linear dynamic range of the calibration equation. The concentration of each HMO was obtained by applying the relevant dilution factors.

**TABLE 1:** MRM transition methods for mass spectrometry measurements of eight HMOs.

HMOs	Molecular weight	Precursor type	m/z			Collision energy
			Precursor ion	→	Product ion	
2′‐FL	488.44	[M+Na]^+^	513.2	→	367.1211	43.9
3′‐FL	488.44	[M+Na]^+^	513.2	→	367.1211	43.9
3′‐SL	633.55	[M–H]^–^	634.4	→	290.0890	−40.5
6′‐SL	633.55	[M–H]^–^	634.4	→	290.0890	−40.5
LSTa	998.88	[M–H]^–^	999.4	→	290.0890	−61.3
LSTb	998.88	[M–H]^–^	999.4	→	290.0890	−61.3
LSTc	998.88	[M–H]^–^	999.4	→	290.0890	−61.3
DSLNT	1290.16	[M–2H]^2–^	644.7	→	999.0350	−35.7

Abbreviations: DSLNT, disialyllacto‐*N*‐tetraose; 2′‐FL, 2′‐fucosyllactose; 3′‐FL, 3′‐fucosyllactose; HMOs, human milk oligosaccharides; LSTa, lactosialyl‐tetrasaccharide a; LSTb, lactosialyl‐tetrasaccharide b; LSTc, lactosialyltetrasaccharide c; MRM, multiple reaction monitoring; 3′‐SL, 3′‐sialyllactose; 6′‐SL, 6′‐sialyllactose.

### 2.5 Method validation

For method validation, we evaluated the selectivity, linearity, range, precision, and accuracy of the quantification methods.

To evaluate selectivity, the separation of HMO isomers was assessed. The resolution (Rs) was calculated to confirm that each HMO isomer was sufficiently separated in the chromatographic analysis. An analysis of 5 pmol of each HMO standard was used for the Rs calculation. The degree of separation was calculated using the following equation. Here, the criterion for sufficient separation was set to Rs > 1.5.

$$\mathrm{Rs}=1.18\times \left(T_{R2}-T_{R1}\right)/\left(W_{0.5h1}+W_{0.5h2}\right)$$

T_R1_, T_R2_: Retention time for each peak (T_R1_ ≤ T_R2_)

W_0.5h1_, W_0.5h2_: Half‐width of each peak

For the evaluation of linearity and range, we constructed standard curves for each HMO standard. Each HMO standard was measured at concentrations ranging from 0.15625 to 10 pmol/µL. The quantification range was defined as any range where the back‐calculated value of each measurement fell within 80–120% of the theoretical value. A coefficient of determination (*R* ^2^) > 0.98 was set as the acceptable linearity criterion.

Precision of the methods was assessed on both within‐ and between‐days. To evaluate within‐day precision, each HMO level in SRM1953 was independently analyzed six times on the same day. For between‐day precision, each HMO level in SRM1953 was analyzed in duplicate over 6 independent days. Precision was evaluated by the relative standard deviation (RSD) in both within‐ (*n* = 6) and between‐ (*n* = 2 × 6) days.

Method accuracy was evaluated through spike and recovery tests. Known amounts of HMOs were spiked into bovine milk at three different levels. Each spiked and nonspiked sample was independently analyzed in triplicate. Recoveries were calculated by subtracting the amount of HMOs measured in the nonspiked sample from the amount measured in the spiked sample and comparing it to the known amount of spiked HMOs.

### 2.6 Demographic data and breast milk

The evaluation of the eight HMOs in breast milk was conducted as a secondary analysis of our cohort data (Saito et al., 2023), based on data obtained from the TMM BirThree Cohort Study (Kuriyama et al., 2020). A total of 150 mother–infant pairs were randomly selected from participants included in the TMM BirThree cohort. Figure S1 depicts the flowchart and the inclusion criteria for the random selection of participants. Participants with < 0.1 mg/mL of 2′‐FL in breast milk were classified as low secretors.

This study adhered to the “Ethical guidelines for human genome and gene analysis research” presented by the Ministry of Education, Culture, Sports, Science and Technology (MEXT); Ministry of Health, Labour and Welfare (MHLW); and Ministry of Economy, Trade, and Industry, as well as the “Ethical guidelines for medical and health research involving human subjects” presented by the MEXT and MHLW. All procedures were approved by the Tohoku University Tohoku Medical Megabank Organization Research Ethics Review Board (approved number: 2024‐4‐016). The use of breast milk and background data reposited in the integrated biobank was approved by the sample and data access committee of the Tohoku Medical Megabank Project. Prior to study initiation, detailed information regarding the study was disclosed on the website of the Tohoku Medical Megabank Organization to allow participants the opportunity to refuse participation.

Breast milk samples collected at 1‐month postpartum were obtained from the TMM‐integrated biobank. The detail information of the biobank has been described by Minegishi et al. (2019). The participants in the TMM BirThree cohort were 22,493 mothers and 23,143 infants, and 458 pairs met the inclusion criteria for this study (Figure S1). Among them, 150 breast milk samples were randomly selected along with demographic data. The lactation stage of the 150 breast milk samples was 32 days (median).

### 2.7 Growth of HC and neurodevelopmental outcomes

HC measurements were obtained from the medical records of the TMM BirThree cohort at birth and at 1, 5, and 9 months of age. These periods were defined as 0, 15–44, 120–179, and 240–299 days, respectively. In cases where multiple HC measurements were recorded within any 1 evaluation month, the dataset with the earlier consultation date was used for the statistical analysis. HC at birth and at 1, 5, and 9 months was standardized into z‐scores (HCZ) based on WHO growth standards (World Health Organization, 2006). The ΔHCZ at 0–1, 0–5, and 0–9 months was calculated by subtracting HCZ at birth from HCZ at 1, 5, and 9 months, respectively.

Neurodevelopmental outcomes at 6, 12, and 24 months of age were also obtained from the TMM BirThree cohort. In the cohort study, neurodevelopmental outcomes were assessed using the ASQ‐3. The ASQ‐3 is a validated screening questionnaire used to evaluate infants and toddlers for the risk of developmental delay across five developmental domains: communication skills, gross motor skills, fine motor skills, problem‐solving ability, and personal and social skills (Mezawa et al., 2019). Each domain is evaluated using six questions designed to assess the attainment of relevant skills. Scores for each item are summed to obtain the overall score for each of the five domains, with a potential range of 0–60 points in 5‐point increments, following the guidance of the Japanese version of the ASQ‐3 (Jane Squires et al., 2021).

### 2.8 Statistical analyses

The association between ΔHCZ and each HMO was screened by Pearson's correlation coefficient. Similarly, the association between ASQ‐3 scores, treated as nonparametric data, and each HMO was screened using Spearman's rank correlation coefficient.

To further investigate the association between ΔHCZ and each HMO level in breast milk, multiple regression analysis was conducted to calculate the coefficient (β) and 95% confidence interval (CI). This analysis focused on potential associations between ΔHCZ and each HMO (*p* < 0.1) identified through Pearson's correlation coefficient. The multiple regression analysis was adjusted by gestational age at birth, household income, maternal alcohol consumption during pregnancy, and maternal passive smoking during pregnancy. Infants whose HC data did not align with the age definition in months for evaluation were excluded from the statistical analysis.

Similarly, to further assess the association between ASQ‐3 scores and each HMO level in breast milk, ordinal logistic regression analysis was performed to calculate the common odds ratio (cOR) and 95% CI values. This analysis focused on potential associations between ASQ‐3 scores and each HMO (*p* < 0.1) identified through Spearman's rank correlation coefficient. In the ordinal logistic regression analysis, ASQ‐3 scores were categorized into 13 levels (0, 5, 10, 15, 20, 25, 30, 35, 40, 45, 50, 55, and 60) due to the score range of 0–60 in 5‐point increments.

Mother–infant pairs with missing background data were excluded from each multivariate analysis. Breast milk samples in which the 2′‐FL level fell below the calibration curve were treated as 0 mg/mL. In multiple regression analysis, a 95% CI of β that did not cross 0 (*p* < 0.05) was considered statistically correlated. Similarly, in ordinal logistic regression analysis, a 95% CI of cOR that did not cross 1 (*p* < 0.05) was considered statistically correlated. In other statistical analysis, a *p*‐value < 0.05 was considered statistically significant. We aimed to study the associations between HMOs levels in breast milk and infant outcomes in an exploratory manner. Therefore, multivariant models were not adjusted for multiple comparisons and were considered exploratory. All statistical analyses were conducted using R statistical software version 4.1.3 (The R Foundation for Statistical Computing) and BellCurve for Excel (version 3.20).

## 3 RESULTS

### 3.1 Development and validation of absolute quantification methods for eight HMOs

**FIGURE 2:**
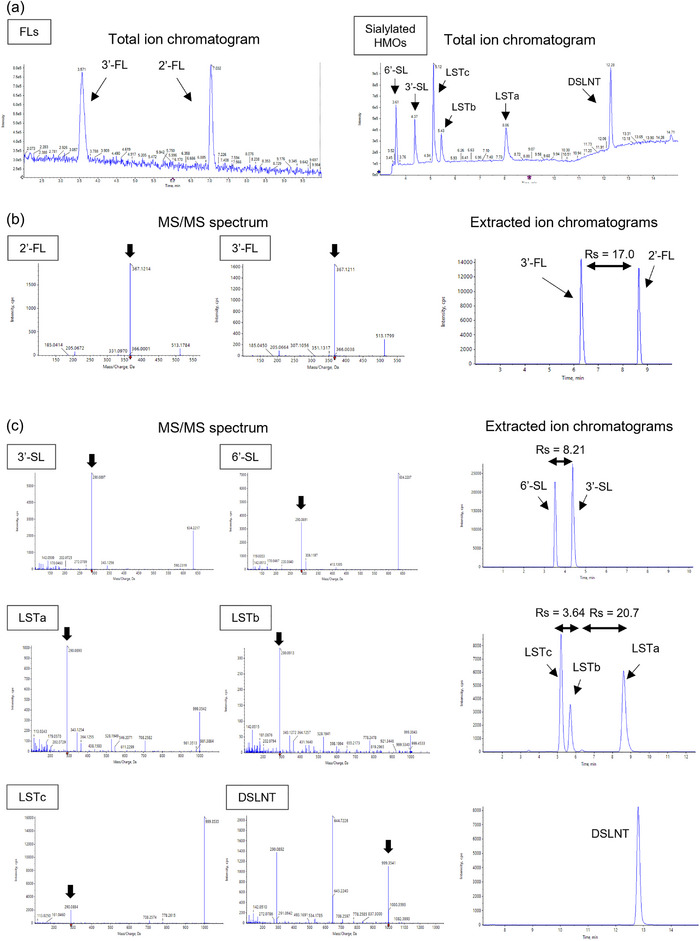
Chromatograms and MS/MS spectra of 2′‐/3′‐fucosyllactose (FLs) and sialylated HMO measurements. (A) Total ion chromatograms of FLs and sialylated HMOs measurements. (B) MS/MS spectrum and extracted ion chromatograms of sialylated HMO measurements. (C) MS/MS spectrum and extracted ion chromatograms of sialylated HMO measurements. Each chromatogram was obtained from the analysis of 5 pmol of HMO standards. DSLNT, disialyllacto‐*N*‐tetraose; FLs, fucosyllactose; 2′‐FL, 2′‐fucosyllactose; 3′‐FL, 3′‐fucosyllactose; HMOs, human milk oligosaccharides; LSTa, lactosialyl‐tetrasaccharide a; LSTb, lactosialyl‐tetrasaccharide b; LSTc, lactosialyltetrasaccharide c; Rs, resolution; 3′‐SL, 3′‐sialyllactose; 6′‐SL, 6′‐sialyllactose.

To develop the quantification method of eight HMOs, we evaluated selectivity to confirm the separation of HMO isomer pairs using 5 pmol of each HMOs standard. The total ion chromatograms for FLs and sialylated HMOs measurements are shown in Figure 2A. The MS/MS spectra and extracted ion chromatograms for FLs and sialylated HMOs are shown in Figure 2B,C. All isomer pairs (2′‐FL and 3′‐FL; 3′‐SL and 6′‐SL; and LSTa, LSTb, and LSTc) were well‐separated in extracted ion chromatograms (Rs > 1.5). In the standard reference material of breast milk (SRM1953), each HMO and all isomer pairs were well selected and separated (Figure S2). Therefore, subsequent evaluations were performed in this chromatographic method.

Following the selectivity evaluation, we determined the range for quantification and evaluated the linearity. To improve the accuracy at lower concentrations, we fitted a linear model with 1/x weighting. The linearity of each HMO standard was evaluated in the range of 1.5625–10 pmol/µL, and the results are shown in Table 2. The plots for the evaluation of linearity are shown in Figure S3, indicating that none of the HMO calibration curves could include higher concentrations (> 10 pmol/µL) because the intensity tended to be suppressed. The calibration curve of each HMO, except 3′‐FL, showed high linearity (*R* ^2^ > 0.99) in any range below the following: 2′‐FL, 0.625–10 pmol/µL; 3′‐SL, 0.15625–5 pmol/µL; 6′‐SL, 0.3125–5 pmol/µL; LSTa, 0.15625–5 pmol/µL; LSTb, 0.15625–10 pmol/µL; LSTc, 0.15625–5 pmol/µL; and DSLNT, 0.3125–10 pmol/µL. The calibration curve of 3′‐FL levels had moderate linearity (*R* ^2^ > 0.98) in the range of 1.5625–5 pmol/µL. The subsequent measurements were performed in the ranges listed in Table 2. At the lowest concentration (0.15,625 pmol/µL), all HMOs were detected above signal‐to‐noise ratio (S/N) > 10 (Table 2). Typically, an S/N > 10 is acceptable for the limit of quantification (LoQ) (ICH harmonised guideline, 2023). Therefore, the range of each HMO in this study was considered above the LoQ.

**TABLE 2:** Linearity and range of HMO measurements.

HMOs	Regression equation (weighting 1/x)	min S/N^*^	Accuracy (%), *n* = 2							*R* ^2^	Range (pmol/µL)
			pmol/µL								
			0.15625	0.3125	0.625	1.25	2.5	5	10		
2′‐FL	y = 13,340.1x – 5566.0	125.5	–	–	104.5	95.0	96.7	105.4	98.5	0.9975	0.625–10
3′‐FL	y = 18,569.6x – 2070.4	209.3	109.8	83.2	97.8	101.2	114.5	93.5	–	0.9861	0.15625–5
3′‐SL	y = 19,883.7x + 629.2	695.9	80.4	100.9	110.3	111.1	101.5	95.8	–	0.9941	0.15625–5
6′‐SL	y = 15,558.6x + 1143.9	555.7	–	83.6	106.1	110.4	104.5	95.4	–	0.9920	0.3125–5
LSTa	y = 23,894.4x – 366.2	576.5	89.1	110.0	98.4	101.4	103.1	98.0	–	0.9980	0.15625–5
LSTb	y = 9387.9x – 55.2	122.0	90.3	97.4	101.2	106.8	101.9	107.3	95.2	0.9956	0.15625–10
LSTc	y = 13,939.1x – 768.3	479.2	94.6	100.6	100.2	105.2	101.2	98.2	–	0.9986	0.15625–5
DSLNT	y = 14,885.3x + 2032.2	589.9	–	82.9	101.6	107.8	111.4	99.6	96.8	0.9935	0.3125–10

Abbreviations: DSLNT, disialyllacto‐*N*‐tetraose; 2′‐FL, 2′‐fucosyllactose; 3′‐FL, 3′‐fucosyllactose; HMOs, human milk oligosaccharides;LSTa, lactosialyl‐tetrasaccharide a; LSTb, lactosialyl‐tetrasaccharide b; LSTc, lactosialyltetrasaccharide c; S/N; signal‐to‐noise ratio; 3′‐SL, 3′‐sialyllactose; 6′‐SL, 6′‐sialyllactose.

* min S/N, the lower S/N in the duplicate measurements at 0.15,625 pmol/µL.

Precision was evaluated by repeated measurements of HMOs in SRM1953 on the within‐ or between‐day (*n* = 6 and *n* = 2 × 6 respectively; Table 3). The most variable precision on the within‐day was RSD = 9.8% for DSLNT. The most variable precision on the between‐day was RSD = 12.7% for LSTc.

**TABLE 3:** Precision of HMO measurements.

HMOs	Within‐day, *n* = 6		Between‐day, *n* = 2 × 6	
	Ave ± SD (mg/mL)	RSD	Ave ± SD (mg/mL)	RSD
2′‐FL	1.34 ± 0.05	4.0%	1.27 ± 0.15	11.9%
3′‐FL	0.80 ± 0.05	6.2%	0.76 ± 0.08	10.0%
3′‐SL	0.16 ± 0.01	5.1%	0.15 ± 0.02	11.9%
6′‐SL	0.11 ± 0.01	5.9%	0.11 ± 0.01	9.4%
LSTa	0.0094 ± 0.0003	3.2%	0.0093 ± 0.0009	9.2%
LSTb	0.062 ± 0.001	2.0%	0.060 ± 0.005	8.2%
LSTc	0.067 ± 0.005	7.9%	0.064 ± 0.008	12.7%
DSLNT	0.19 ± 0.02	9.8%	0.17 ± 0.02	12.4%

*Note*: Standard reference material of breast milk (SRM1953; National Institute of Standards and Technology, USA) was used to evaluate the precision of the quantifications.

Abbreviations: DSLNT, disialyllacto‐*N*‐tetraose; 2′‐FL, 2′‐fucosyllactose; 3′‐FL, 3′‐fucosyllactose; HMOs, human milk oligosaccharides; LSTa, lactosialyl‐tetrasaccharide a; LSTb, lactosialyl‐tetrasaccharide b; LSTc, lactosialyltetrasaccharide c; 3′‐SL, 3′‐sialyllactose; 6′‐SL, 6′‐sialyllactose.

To confirm the accuracy of values from the methods, a spike and recovery test using bovine milk was performed (Table 4). The lowest accuracy was observed for 6′‐SL at the high spike level, which was 79.4%. In other HMOs and each spike level test, the accuracy was 80.5–110.9%.

**TABLE 4:** Spike and recovery test (*n* = 3 each).

HMOs	Original conc. (pmol/µL)	Low			Moderate			High		
		Spiked (+ pmol/µL)	Measured (+ pmol/µL)	Average recovery	Spiked (+ pmol/µL)	Measured (+ pmol/µL)	Average recovery	Spiked (+ pmol/µL)	Measured (+ pmol/µL)	Average recovery
2′‐FL	N.D.	500	402.4 ± 19.1	80.5%	1000	817.9 ± 45.9	81.8%	2000	1870.8 ± 330.5	93.5%
3′‐FL	3.3 ± 0.8	500	434.7 ± 24.9	86.3%	1000	974.2 ± 22.8	97.1%	2000	1848.0 ± 303.1	92.2%
3′‐SL	77.9 ± 10.2	200	288.6 ± 6.2	105.4%	500	487.5 ± 23.8	81.9%	1000	889.6 ± 35.3	81.2%
6′‐SL	15.2 ± 0.9	200	192.7 ± 4.5	88.7%	500	440.4 ± 18.7	85.0%	1000	809.4 ± 3.4	79.4%
LSTa	N.D.	10	11.1 ± 0.3	110.9%	25	27.0 ± 0.6	108.2%	100	91.8 ± 2.9	91.8%
LSTb	N.D.	25	26.3 ± 0.7	105.2%	100	98.0 ± 2.9	98.0%	200	183.4 ± 4.2	91.7%
LSTc	N.D.	25	26.8 ± 1.9	107.2%	100	94.1 ± 2.8	94.1%	200	167.6 ± 1.3	83.8%
DSLNT	N.D.	100	94.3 ± 2.0	94.3%	250	249.7 ± 14.2	99.9%	500	425.8 ± 21.3	85.2%

*Note*: Original conc. shows the concentration of each HMO in bovine milk. N.D. indicates that no analytes were detected above the minimum concentration of the standard curves (> 0.15,625 pmol/µL).

Abbreviations: DSLNT, disialyllacto‐N‐tetraose; 2′‐FL, 2′‐fucosyllactose; 3′‐FL, 3′‐fucosyllactose; HMOs, human milk oligosaccharides; LSTa, lactosialyl‐tetrasaccharide a; LSTb, lactosialyl‐tetrasaccharide b; LSTc, lactosialyltetrasaccharide c; 3′‐SL, 3′‐sialyllactose; 6′‐SL, 6′‐sialyllactose.

### 3.2 HMO profiles in breast milk of the Tohoku Medical Megabank Project Birth and Three‐Generation Cohort Study

**FIGURE 3:**
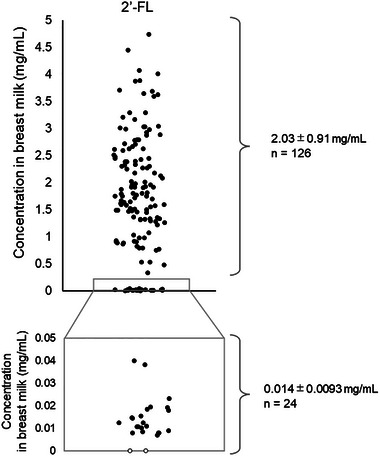
Dot plot of the 2′‐fucosyllactose (2′‐FL) levels in breast milk (*n* = 150). White circles (°) represent breast milk categorized as 0 mg/mL, as no 2′‐FL was detected above the minimum concentration of the standard curves.

**FIGURE 4:**
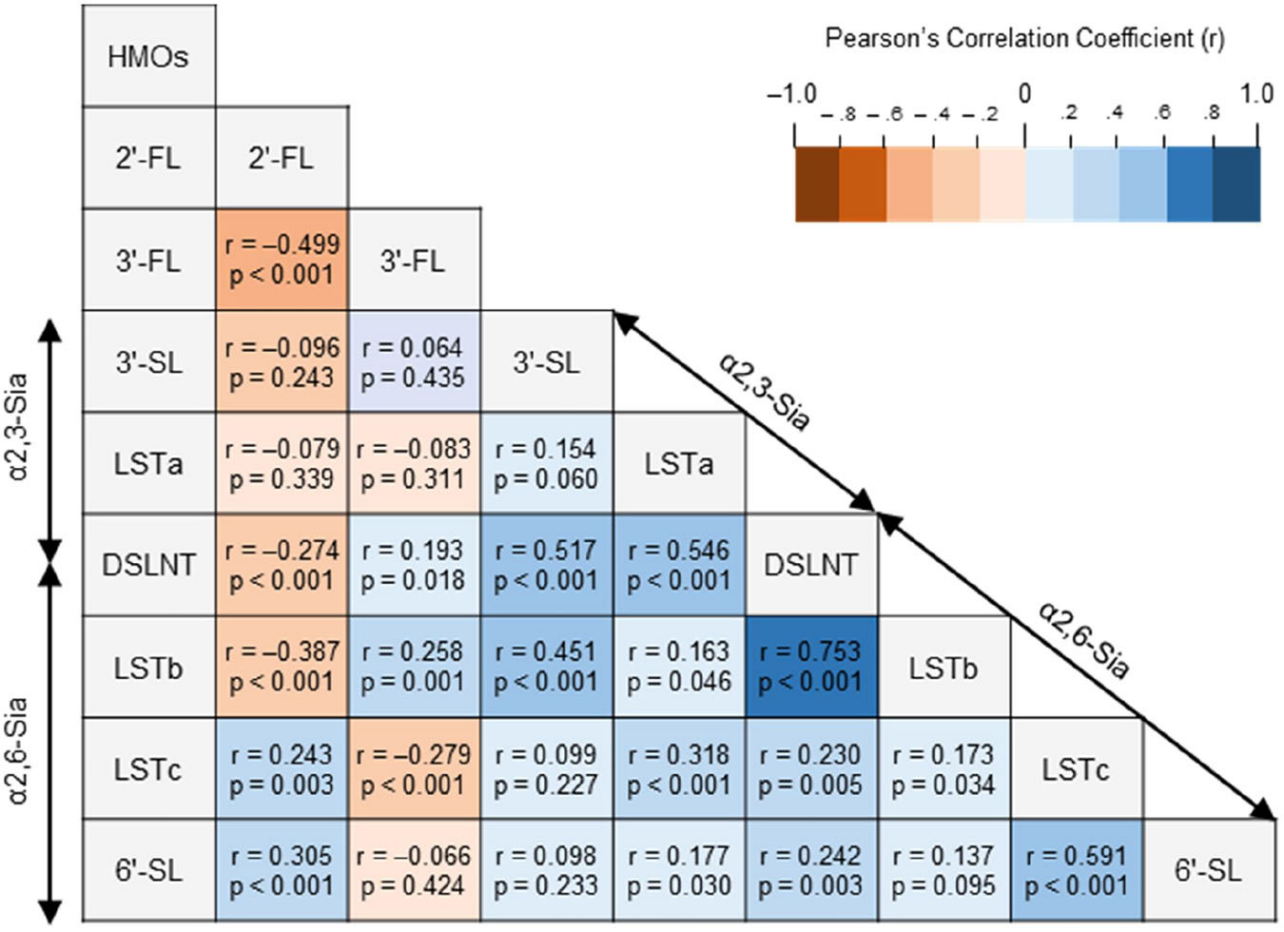
Correlogram of eight HMO levels in breast milk. Pearson's correlation coefficients (*r*) and *p*‐values are shown. DSLNT, disialyllacto‐*N*‐tetraose; 2′‐FL, 2′‐fucosyllactose; 3′‐FL, 3′‐fucosyllactose; HMOs, human milk oligosaccharides; LSTa, lactosialyl‐tetrasaccharide a; LSTb, lactosialyl‐tetrasaccharide b; LSTc, lactosialyltetrasaccharide c; 3′‐SL, 3′‐sialyllactose; 6′‐SL, 6′‐sialyllactose.

Following the development of quantification methods, eight HMOs were quantified in 1‐month breast milk samples selected from the TMM BirThree Cohort Study (*n* = 150). The demographic data of infant–mother pairs in secretors and low secretors are presented in Table S1. FL measurements indicated that 24 out of 150 samples were estimated to be low secretors, exhibiting extremely low 2′‐FL levels (< 0.1 mg/mL, Figure 3). Evaluation using Pearson's correlation coefficient revealed concentration correlations among several individual HMO levels (Figure 4). Specifically, the level of 2′‐FL in breast milk showed negative correlations with 3′‐FL (*r* = –0.499, *p* < 0.001), LSTb (*r* = –0.387, *p* < 0.001), and DSLNT (*r* = –0.274, *p* < 0.001), as well as positive correlations with 6′‐SL (*r* = 0.305, *p* < 0.001) and LSTc (*r* = 0.243, *p* = 0.003). Positive correlations were also observed mainly among sialylated HMOs with the same sialic acid linkage: α2,3‐linked sialic acids (between 3′‐SL and DSLNT [*r* = 0.517, *p* < 0.001] and between LSTa and DSLNT [*r* = 0.546, *p* < 0.001]) and α2,6‐linked sialic acid (between LSTb and DSLNT [*r* = 0.753, *p* < 0.001], between LSTc and DSLNT [*r* = 0.230, *p* < 0.005], between 6′‐SL and DSLNT [*r* = 0.242, *p* = 0.003], between LSTb and LSTc [*r* = 0.173, *p* = 0.034], and between 6′‐SL and LSTc [*r* = 0.591, *p* < 0.001]).

The concentrations of the eight HMOs in the breast milk of secretors and low secretors are shown in Table 5. Significant differences were observed between secretors and low secretors for 3′‐FL, LSTb, LSTc, and DSLNT levels, in addition to 2′‐FL levels.

**TABLE 5:** HMO concentration in 1‐month Japanese breast milk.

HMOs	Low secretor (*n* = 24)	Secretor (*n* = 126)	*p*‐value^a^
	Ave ± SD (mg/mL)	Ave ± SD (mg/mL)	
2′‐FL	0.014 ± 0.009	2.03 ± 0.91	*p* < 0.001
3′‐FL	0.80 ± 0.41	0.29 ± 0.18	*p* < 0.001
3′‐SL	0.15 ± 0.05	0.15 ± 0.04	0.479
6′‐SL	0.33 ± 0.12	0.36 ± 0.13	0.338
LSTa	0.015 ± 0.008	0.013 ± 0.007	0.250
LSTb	0.14 ± 0.07	0.092 ± 0.050	*p* < 0.001
LSTc	0.10 ± 0.04	0.14 ± 0.08	0.011
DSLNT	0.35 ± 0.15	0.26 ± 0.11	*p* < 0.001

Abbreviations: DSLNT, disialyllacto‐*N*‐tetraose; 2′‐FL, 2′‐fucosyllactose; 3′‐FL, 3′‐fucosyllactose; HMOs, human milk oligosaccharides; LSTa, lactosialyl‐tetrasaccharide a; LSTb, lactosialyl‐tetrasaccharide b; LSTc, lactosialyltetrasaccharide c; 3′‐SL, 3′‐sialyllactose; 6′‐SL, 6′‐sialyllactose.

^a^ The *p*‐values are for the differences among participants between the low secretor and secretor groups using *t*‐tests.

### 3.3 Association between neurodevelopmental outcomes and each HMO level in breast milk

We evaluated the association between each HMO level in breast milk and head growth (ΔHCZ) during infancy, as well as subsequent neurodevelopmental scores (ASQ‐3), in an exploratory manner. The collected data of ΔHCZ at 0–1, 0–5, and 0–9 months and ASQ‐3 scores at 6, 12, and 24 months are presented in Tables S2 and S3 (separately shown for secretors and low secretors). To screen for positive associations between each outcome and each HMO level, an exploratory analysis was performed using Pearson's correlation coefficient for ΔHCZ and Spearman's rank coefficient correlation for the ASQ‐3 scores among all (Table 6) and secretor participants (Table 7). Among all participants, only 2′‐FL levels in breast milk showed positive associations for both ΔHCZ (at 0–5 months: *r* = 0.198, *p* = 0.019) and ASQ‐3 scores (fine motor at 24 months: ρ = 0.163, *p* = 0.047) (Table 6). Among secretors, the association between each HMO and each outcome considerably changed from the association among all participants (Table 7). Although the analysis of all participants did not suggest a positive association between the sialylated HMO levels and ASQ‐3 scores, exploratory evaluation among secretors suggested a positive association between some sialylated HMO levels and ASQ‐3 scores (between gross motor at 12 months and LSTb levels: ρ = 0.195, *p* = 0.029; between gross motor at 24 months and DSLNT levels: ρ = 0.180, *p* = 0.043). Tables S4 and S5 provide the exploratory analysis for associations among low secretor participants and that between grouped HMOs, respectively.

**TABLE 6:** Association between eight HMO levels in breast milk and changes in head circumference (ΔHCZ) or neurodevelopmental scores (Age and Stages Questionnaire, Third Edition [ASQ‐3]) among all participants.

	HMOs							
	2′‐FL	3′‐FL	3′‐SL	6′‐SL	LSTa	LSTb	LSTc	DSLNT
ΔHCZ								
0–1 month (*n* = 121)	*r* = 0.057 *p* = 0.536	*r* = 0.102 *p* = 0.267	*r* = 0.080 *p* = 0.381	*r* = 0.002 *p* = 0.985	*r* = 0.004 *p* = 0.961	*r* = 0.174 *p* = 0.057†	*r* = –0.027 *p* = 0.768	*r* = 0.177 *p* = 0.052†
0–5 months (*n* = 139)	*r* = 0.198 *p* = 0.019*	*r* = –0.021 *p* = 0.804	*r* = –0.136 *p* = 0.112	*r* = 0.023 *p* = 0.789	*r* = 0.076 *p* = 0.373	*r* = –0.040 *p* = 0.644	*r* = 0.098 *p* = 0.250	*r* = –0.022 *p* = 0.798
0–9 months (*n* = 135)	*r* = 0.120 *p* = 0.166	*r* = 0.060 *p* = 0.492	*r* = –0.148 *p* = 0.087†	*r* = –0.034 *p* = 0.696	*r* = 0.045 *p* = 0.602	*r* = –0.042 *p* = 0.628	*r* = 0.018 *p* = 0.832	*r* = –0.002 *p* = 0.977
ASQ‐3 scores								
At 6 months (*n* = 150)								
Communication	ρ = –0.027 *p* = 0.746	ρ = 0.071 *p* = 0.391	ρ = –0.103 *p* = 0.208	ρ = –0.068 *p* = 0.409	ρ = –0.064 *p* = 0.439	ρ = –0.156 *p* = 0.057 †	ρ = –0.053 *p* = 0.516	ρ = –0.082 *p* = 0.321
Gross motor	ρ = –0.015 *p* = 0.855	ρ = –0.012 *p* = 0.886	ρ = –0.026 *p* = 0.750	ρ = –0.049 *p* = 0.554	ρ = –0.023 *p* = 0.784	ρ = –0.022 *p* = 0.789	ρ = 0.013 *p* = 0.874	ρ = –0.013 *p* = 0.871
Fine motor	ρ = –0.106 *p* = 0.199	ρ = 0.084 *p* = 0.306	ρ = 0.002 *p* = 0.980	ρ = –0.053 *p* = 0.520	ρ = 0.055 *p* = 0.502	ρ = 0.027 *p* = 0.740	ρ = –0.049 *p* = 0.552	ρ = 0.027 *p* = 0.743
Problem‐solving	ρ = –0.034 *p* = 0.682	ρ = –0.007 *p* = 0.931	ρ = –0.011 *p* = 0.896	ρ = –0.061 *p* = 0.459	ρ = 0.020 *p* = 0.805	ρ = –0.052 *p* = 0.531	ρ = –0.013 *p* = 0.877	ρ = 0.0003 *p* = 0.997
Personal–social	ρ = –0.012 *p* = 0.886	ρ = –0.031 *p* = 0.706	ρ = –0.085 *p* = 0.301	ρ = –0.109 *p* = 0.186	ρ = –0.083 *p* = 0.311	ρ = –0.142 *p* = 0.082†	ρ = –0.035 *p* = 0.670	ρ = –0.119 *p* = 0.147
At 12 months (*n* = 150)								
Communication	ρ = 0.0004 *p* = 0.996	ρ = –0.033 *p* = 0.693	ρ = –0.029 *p* = 0.727	ρ = –0.016 *p* = 0.847	ρ = –0.056 *p* = 0.497	ρ = 0.072 *p* = 0.384	ρ = 0.041 *p* = 0.614	ρ = 0.031 *p* = 0.708
Gross motor	ρ = 0.007 *p* = 0.935	ρ = –0.043 *p* = 0.602	ρ = 0.108 *p* = 0.189	ρ = 0.094 *p* = 0.253	ρ = 0.016 *p* = 0.849	ρ = 0.100 *p* = 0.221	ρ = 0.007 *p* = 0.935	ρ = 0.092 *p* = 0.262
Fine motor	ρ = –0.012 *p* = 0.884	ρ = 0.030 *p* = 0.715	ρ = 0.024 *p* = 0.770	ρ = 0.067 *p* = 0.419	ρ = –0.016 *p* = 0.844	ρ = 0.055 *p* = 0.505	ρ = –0.002 *p* = 0.980	ρ = 0.040 *p* = 0.631
Problem‐solving	ρ = –0.034 *p* = 0.681	ρ = 0.014 *p* = 0.870	ρ = –0.001 *p* = 0.986	ρ = –0.059 *p* = 0.474	ρ = –0.062 *p* = 0.453	ρ = 0.054 *p* = 0.515	ρ = –0.041 *p* = 0.618	ρ = –0.031 *p* = 0.709
Personal–social	ρ = 0.033 *p* = 0.688	ρ = –0.032 *p* = 0.701	ρ = –0.008 *p* = 0.924	ρ = –0.090 *p* = 0.275	ρ = –0.035 *p* = 0.668	ρ = –0.002 *p* = 0.983	ρ = –0.002 *p* = 0.977	ρ = 0.001 *p* = 0.991
At 24 months (*n* = 150)								
Communication	ρ = –0.036 *p* = 0.665	ρ = –0.015 *p* = 0.852	ρ = –0.131 *p* = 0.110	ρ = –0.103 *p* = 0.209	ρ = –0.053 *p* = 0.520	ρ = –0.078 *p* = 0.344	ρ = –0.137 *p* = 0.095†	ρ = –0.070 *p* = 0.392
Gross motor	ρ = 0.020 *p* = 0.809	ρ = 0.033 *p* = 0.688	ρ = –0.053 *p* = 0.520	ρ = 0.025 *p* = 0.757	ρ = 0.113 *p* = 0.170	ρ = 0.054 *p* = 0.515	ρ = –0.014 *p* = 0.866	ρ = 0.130 *p* = 0.112
Fine motor	ρ = 0.163 *p* = 0.047*	ρ = –0.089 *p* = 0.281	ρ = 0.011 *p* = 0.893	ρ = 0.007 *p* = 0.933	ρ = 0.019 *p* = 0.821	ρ = –0.109 *p* = 0.185	ρ = 0.009 *p* = 0.913	ρ = –0.024 *p* = 0.772
Problem‐solving	ρ = 0.067 *p* = 0.417	ρ = –0.010 *p* = 0.904	ρ = –0.057 *p* = 0.489	ρ = 0.064 *p* = 0.440	ρ = 0.004 *p* = 0.965	ρ = –0.085 *p* = 0.301	ρ = 0.052 *p* = 0.530	ρ = –0.017 *p* = 0.840
Personal–social	ρ = 0.059 *p* = 0.474	ρ = –0.006 *p* = 0.947	ρ = –0.112 *p* = 0.173	ρ = –0.039 *p* = 0.632	ρ = –0.150 *p* = 0.066†	ρ = –0.072 *p* = 0.379	ρ = –0.028 *p* = 0.733	ρ = –0.145 *p* = 0.076†

Abbreviations: DSLNT, disialyllacto‐*N*‐tetraose; 2′‐FL, 2′‐fucosyllactose; 3′‐FL, 3′‐fucosyllactose; HMOs, human milk oligosaccharides; LSTa, lactosialyl‐tetrasaccharide a; LSTb, lactosialyl‐tetrasaccharide b; LSTc, lactosialyltetrasaccharide c; 3′‐SL, 3′‐sialyllactose; 6′‐SL, 6′‐sialyllactose.

*r*, Pearson's correlation coefficient; ρ, Spearman's rank correlation coefficient; *, *p* < 0.05; †, *p* < 0.1.

**TABLE 7:** Association between eight HMO levels in breast milk and changes in head circumference (ΔHCZ) or neurodevelopmental scores (Age and Stages Questionnaire, Third Edition [ASQ‐3]) among secretor participants.

	HMOs							
	2′‐FL	3′‐FL	3′‐SL	6′‐SL	LSTa	LSTb	LSTc	DSLNT
ΔHCZ								
0–1 month (*n* = 103)	*r* = 0.167 *p* = 0.092†	*r* = –0.038 *p* = 0.703	*r* = 0.005 *p* = 0.956	*r* = 0.052 *p* = 0.599	*r* = 0.041 *p* = 0.684	*r* = 0.107 *p* = 0.283	*r* = –0.005 *p* = 0.958	*r* = 0.158 *p* = 0.110
0–5 months (*n* = 117)	*r* = 0.229 *p* = 0.013*	*r* = 0.048 *p* = 0.606	*r* = –0.162 *p* = 0.081†	*r* = 0.017 *p* = 0.858	*r* = 0.053 *p* = 0.569	*r* = 0.034 *p* = 0.720	*r* = 0.098 *p* = 0.293	*r* = 0.009 *p* = 0.925
0–9 months (*n* = 116)	*r* = 0.192 *p* = 0.039*	*r* = –0.028 *p* = 0.763	*r* = –0.183 *p* = 0.049*	*r* = –0.001 *p* = 0.992	*r* = 0.091 *p* = 0.330	*r* = –0.027 *p* = 0.771	*r* = 0.045 *p* = 0.628	*r* = –0.025 *p* = 0.791
ASQ‐3 Scores								
At 6 months (*n* = 126)								
Communication	ρ = –0.059 *p* = 0.514	ρ = 0.034 *p* = 0.702	ρ = –0.098 *p* = 0.277	ρ = –0.080 *p* = 0.371	ρ = –0.023 *p* = 0.794	ρ = –0.140 *p* = 0.118	ρ = –0.035 *p* = 0.699	ρ = –0.051 *p* = 0.571
Gross motor	ρ = –0.146 *p* = 0.103	ρ = 0.078 *p* = 0.383	ρ = 0.030 *p* = 0.738	ρ = –0.095 *p* = 0.288	ρ = 0.007 *p* = 0.937	ρ = 0.036 *p* = 0.689	ρ = –0.060 *p* = 0.506	ρ = 0.075 *p* = 0.403
Fine motor	ρ = –0.092 *p* = 0.306	ρ = 0.049 *p* = 0.587	ρ = 0.085 *p* = 0.342	ρ = –0.059 *p* = 0.511	ρ = 0.088 *p* = 0.328	ρ = 0.036 *p* = 0.687	ρ = –0.073 *p* = 0.419	ρ = 0.083 *p* = 0.355
Problem‐solving	ρ = –0.003 *p* = 0.974	ρ = –0.008 *p* = 0.929	ρ = 0.035 *p* = 0.696	ρ = –0.037 *p* = 0.677	ρ = 0.045 *p* = 0.621	ρ = –0.040 *p* = 0.659	ρ = 0.003 *p* = 0.971	ρ = 0.048 *p* = 0.594
Personal–social	ρ = 0.019 *p* = 0.829	ρ = –0.039 *p* = 0.667	ρ = –0.036 *p* = 0.692	ρ = –0.120 *p* = 0.181	ρ = –0.047 *p* = 0.604	ρ = –0.119 *p* = 0.185	ρ = –0.042 *p* = 0.640	ρ = –0.056 *p* = 0.536
At 12 months (*n* = 126)								
Communication	ρ = –0.066 *p* = 0.464	ρ = 0.008 *p* = 0.928	ρ = 0.008 *p* = 0.926	ρ = –0.057 *p* = 0.528	ρ = –0.060 *p* = 0.506	ρ = 0.112 *p* = 0.212	ρ = –0.035 *p* = 0.700	ρ = 0.070 *p* = 0.438
Gross motor	ρ = –0.137 *p* = 0.125	ρ = 0.079 *p* = 0.381	ρ = 0.159 *p* = 0.076†	ρ = 0.057 *p* = 0.527	ρ = 0.014 *p* = 0.876	ρ = 0.195 *p* = 0.029*	ρ = –0.094 *p* = 0.296	ρ = 0.173 *p* = 0.052†
Fine motor	ρ = –0.112 *p* = 0.213	ρ = 0.129 *p* = 0.149	ρ = 0.106 *p* = 0.236	ρ = 0.080 *p* = 0.375	ρ = 0.010 *p* = 0.915	ρ = 0.167 *p* = 0.062†	ρ = –0.059 *p* = 0.514	ρ = 0.163 *p* = 0.068†
Problem‐solving	ρ = –0.098 *p* = 0.274	ρ = 0.070 *p* = 0.435	ρ = 0.130 *p* = 0.148	ρ = –0.061 *p* = 0.495	ρ = –0.030 *p* = 0.741	ρ = 0.135 *p* = 0.131	ρ = –0.106 *p* = 0.238	ρ = 0.071 *p* = 0.430
Personal–social	ρ = –0.062 *p* = 0.494	ρ = 0.036 *p* = 0.687	ρ = 0.027 *p* = 0.764	ρ = –0.128 *p* = 0.152	ρ = –0.012 *p* = 0.890	ρ = 0.069 *p* = 0.445	ρ = –0.074 *p* = 0.409	ρ = 0.086 *p* = 0.341
At 24 months (*n* = 126)								
Communication	ρ = 0.002 *p* = 0.986	ρ = –0.005 *p* = 0.951	ρ = –0.128 *p* = 0.154	ρ = –0.102 *p* = 0.255	ρ = –0.060 *p* = 0.502	ρ = –0.062 *p* = 0.488	ρ = –0.145 *p* = 0.106	ρ = –0.067 *p* = 0.458
Gross motor	ρ = –0.043 *p* = 0.636	ρ = 0.061 *p* = 0.499	ρ = 0.027 *p* = 0.765	ρ = 0.029 *p* = 0.749	ρ = 0.111 *p* = 0.215	ρ = 0.128 *p* = 0.154	ρ = –0.049 *p* = 0.587	ρ = 0.180 *p* = 0.043*
Fine motor	ρ = 0.052 *p* = 0.560	ρ = 0.017 *p* = 0.852	ρ = 0.093 *p* = 0.301	ρ = –0.031 *p* = 0.732	ρ = 0.001 *p* = 0.988	ρ = –0.050 *p* = 0.577	ρ = –0.082 *p* = 0.359	ρ = 0.023 *p* = 0.795
Problem‐solving	ρ = 0.051 *p* = 0.568	ρ = 0.065 *p* = 0.471	ρ = –0.023 *p* = 0.798	ρ = 0.038 *p* = 0.671	ρ = –0.019 *p* = 0.829	ρ = –0.047 *p* = 0.600	ρ = 0.021 *p* = 0.815	ρ = –0.009 *p* = 0.922
Personal–social	ρ = –0.038 *p* = 0.676	ρ = 0.095 *p* = 0.291	ρ = –0.087 *p* = 0.335	ρ = –0.055 *p* = 0.541	ρ = –0.092 *p* = 0.305	ρ = –0.026 *p* = 0.773	ρ = –0.124 *p* = 0.167	ρ = –0.081 *p* = 0.365

Abbreviations: DSLNT, disialyllacto‐*N*‐tetraose; 2′‐FL, 2′‐fucosyllactose; 3′‐FL, 3′‐fucosyllactose; HMOs, human milk oligosaccharides; LSTa, lactosialyl‐tetrasaccharide a; LSTb, lactosialyl‐tetrasaccharide b; LSTc, lactosialyltetrasaccharide c; 3′‐SL, 3′‐sialyllactose; 6′‐SL, 6′‐sialyllactose.

*r*, Pearson's correlation coefficient; ρ, Spearman's rank correlation coefficient; *, *p* < 0.05; †, *p* < 0.1.

Neurodevelopmental outcomes were influenced by many factors (Benn, 1993). Here, multivariate analyses were performed for combinations of HMOs and outcomes with possible associations (*p* < 0.1) in screening analyses (Tables 8 and 9) to further evaluate the associations between each HMO level in breast milk and infant outcomes by adjustment of potentially confounding factors. ΔHCZ was evaluated using multiple regression analysis, and ASQ‐3 scores were evaluated using ordinal logistic regression analysis. Multivariate analyses were performed among all participants and among the secretor participants. Further evaluation among low secretor participants was not performed due to the small number of participants. Multiple regression analysis among all participants showed positive associations between ΔHCZ at 0–1 month and LSTb levels (β = 3.37, 95% CI = 0.22–6.52) and between ΔHCZ at 0–5 months and 2′‐FL levels (β = 0.21, 95% CI = 0.02–0.40). Among the secretor participants, 2′‐FL levels also showed a positive association with ΔHCZ at 0–5 months (β = 0.33, 95% CI = 0.07–0.59) and ΔHCZ at 0–9 months (β = 0.31, 95% CI = 0.02–0.59). Ordinal logistic regression analysis among all participants showed a positive association between fine motor at 24 months and 2′‐FL levels (cOR = 1.41, 95% CI = 1.06–1.88). Negative associations were observed between personal–social at 6 months and LSTb levels (cOR = 0.48, 95% CI = 0.28–0.81) and between communication at 24 months and LSTc levels (cOR = 0.58, 95% CI = 0.37–0.91). Among the secretor participants, positive correlations were observed between gross motor at 12 months and 3′‐SL levels (cOR = 2.39, 95% CI = 1.05–5.80) and between fine motor at 12 months and DSLNT levels (cOR = 1.45, 95% CI = 1.05–2.02).

**TABLE 8:** Association between HMO levels and changes of head circumference (ΔHCZ) evaluated in multiple regression analysis among all and secretor participants.

ΔHCZ	*n*	β	95% CI	*p‐*values
–HMOs				
All				
At 0–1 month				
—LSTb (mg/mL)	117	3.37	0.22–6.52^*^	0.036
—DSLNT (mg/mL)	117	1.47	−0.04–2.99	0.056
At 0–5 months				
—2′‐FL (mg/mL)	133	0.21	0.02–0.40^*^	0.028
At 0–9 months				
—3′‐SL (mg/mL)	130	−5.17	−10.9–0.51	0.074
Secretors				
At 0–1 month				
—2′‐FL (mg/mL)	99	0.15	−0.09–0.38	0.214
At 0–5 months				
—2′‐FL (mg/mL)	111	0.33	0.07–0.59^*^	0.014
—3′‐SL (mg/mL)	111	−4.87	−10.6–0.83	0.093
At 0–9 months				
—2′‐FL (mg/mL)	111	0.31	0.02–0.59^*^	0.039
—3′‐SL (mg/mL)	111	−5.97	−12.1–0.14	0.055

*Note*: Analysis model was adjusted by gestational age at birth, household income, maternal alcohol consumption during pregnancy, maternal passive smoking during pregnancy, and the number of children in the family.

Abbreviations: DSLNT, disialyllacto‐*N*‐tetraose; 2′‐FL, 2′‐fucosyllactose; HMOs, human milk oligosaccharides; LSTb, lactosialyl‐tetrasaccharide b; 3′‐SL, 3′‐sialyllactose.

* *p* < 0.05.

**TABLE 9:** Association between HMO levels and ASQ‐3 scores evaluated in ordinal logistic regression analysis among all and secretor participants.

ASQ‐3 scores	*n*	cOR	95% CI	*p*‐values
—HMOs				
All				
Communication at 6 M				
—LSTb (× 10 mg/mL)	144	0.80	0.47–1.35	0.407
Personal–social at 6 M				
– LSTb (× 10 mg/mL)	144	0.48	0.28–0.81**	0.006
Communication at 24 M				
—LSTc (× 10 mg/mL)	144	0.58	0.37–0.91*	0.017
Fine motor at 24 M				
—2′‐FL (mg/mL)	144	1.41	1.06–1.88*	0.018
Personal–social at 24 M				
—LSTa (× 10 mg/mL)	144	0.09	0.001–7.04	0.274
—DSLNT (× 10 mg/mL)	144	0.76	0.58–1.002	0.051
Secretors				
Gross motor at 12 M				
—3′‐SL (× 10 mg/mL)	120	2.39	1.05–5.80*	0.044
—LSTb (× 10 mg/mL)	120	1.61	0.84–3.30	0.167
—DSLNT (× 10 mg/mL)	120	1.32	0.96–1.84	0.094
Fine motor at 12 M				
—LSTb (× 10 mg/mL)	120	1.87	0.94–3.82	0.078
—DSLNT (× 10 mg/mL)	120	1.45	1.05–2.02*	0.023
Gross motor at 24 M				
—DSLNT (× 10 mg/mL)	120	1.32	0.96–1.84	0.094

*Note*: Analysis model was adjusted by gestational age at birth, household income, maternal alcohol consumption during pregnancy, maternal passive smoking during pregnancy, and the number of children in the family.

Abbreviations: DSLNT, disialyllacto‐*N*‐tetraose; 2′‐FL, 2′‐fucosyllactose; HMOs, human milk oligosaccharides; LSTb, lactosialyl‐tetrasaccharide b; LSTc, lactosialyltetrasaccharide c; 3′‐SL, 3′‐sialyllactose.

*p < 0.05; **p < 0.01.

## 4 DISCUSSION

Following the development of quantification methods for eight HMOs in breast milk, we applied the developed methods to our TMM BirThree Cohort Study. The results provided the profiles of the eight HMO levels, revealing the ratio of low secretor mothers in Japan and the synchrony of levels in breast milk among some HMOs. Subsequently, we conducted the first study in Japan to evaluate the association between each HMO level in breast milk and neurodevelopmental outcomes in infants in an exploratory manner. The results proposed that head growth was positively associated with LSTb and 2′‐FL levels, whereas certain domains of neurodevelopmental scores were positively associated with 2′‐FL levels in all participants and with 3′‐SL and DSLNT levels in secretors. These quantification methods effectively characterized the HMO levels in Japanese breast milk and provided support for the nutritional association between HMOs and infants’ neurodevelopment.

This study examined the validity of HMO measurement methods in terms of their applicability in clinical research. As standard methods for HMO quantification have not yet been established, validated methods must be developed in each laboratory based on the available equipment. When using the quantification method established in our study, a slight decrease in accuracy was observed for 6′‐SL (high‐level spike, 79.4%; Table 4). This discrepancy may be attributed to the simultaneous removal of HMOs during lipid removal by centrifugation or protein precipitation by ethanol. The precision of the method showed RSD ≤ 12.7% (between‐day; Table 3). The required levels of accuracy and precision for a quantitative method depend on its intended application. The Food and Drug Administration reports that the preferred accuracy and precision are within 15% for clinical applications (The U.S. Food & Drug Administration, 2018). The HMO quantification method using maltotriose calibration by Austin and Bénet (2018) has 86–120% of accuracy and RSD ≤ 15% of precision in intermediate reproducibility (including minor HMOs). The HMO quantification method by Bao et al. (2013) has 86–104% of accuracy and RSD < 9% of precision. Researchers have applied these quantification methods to the evaluation of clinical samples (Austin et al., 2019; Perrin et al., 2017; Samuel et al., 2019, 2022). In this study, one aim of HMO quantification was to evaluate the correlation between HMOs and infant outcomes. The priority was to determine the rank of HMO levels in each breast milk. As high precision allows for reproducible evaluation of large and small HMO levels in breast milk, the method developed in this study is expected to be sufficient for the evaluation of correlation analysis.

However, the method in this study has room for improvement in terms of accuracy. Specifically, the procedure of using internal standards might improve the accuracy of HMO measurements in this study, though the developed method did not use internal standards to simplify the procedure. Xylose, isomaltotriose, and maltotriose are candidates of internal standards based on the methods reported previously (Asakuma et al., 2007, 2008; Austin & Bénet, 2018). Other procedures, such as changes in the analytical scale, might improve the precision of measurements.

There are some limitations with regard to the evaluation of accuracy and the quantification range. First, the matrix of bovine milk, which is used as a negative sample in spike and recovery tests, is different from breast milk. Several studies, including the current study, showed that bovine milk contains considerably lower oligosaccharides than breast milk (Martín‐Sosa et al., 2003; Shi et al., 2021; Zhang et al., 2023; and Supporting Information S1). Therefore, it is expected that bovine milk is suitable as a negative sample in spike and recovery tests. However, the unknown effect of differences in matrix composition from breast milk represents a limitation in this test. Second, a purity test for analytical standards was not performed. The purity of each analytical standard was variable in each product lot, thereby influencing the true value. Therefore, it is possible that the results of this study (e.g., each HMO level in standard breast milk [SRM1953]) may not be reproducible depending on the product lot and/or product of analytical standards used. Third, the narrow quantification range, which is a major weakness of the MRM method, is one of the limitations of this method. The calibration curve plots showed a decrease in response at high concentrations (Figure S3), suggesting that ionization suppression narrowed quantification ranges. Other fitting models can expand the quantification range, although the effects on precision and accuracy remain unknown. While the widest range in this study was 0.15625–10 pmol/µL (Table 2), HPLC with fluorescence labeling methods reported by Austin and Bénet (2018) indicated a much wider range (10–20,000 pmol/µL). The high selectivity of MRM methods represents a powerful strength; however, the HPLC method is probably suitable for measuring large variable HMOs, such as 2′‐FL.

In the evaluation of breast milk samples of the TMM BirThree cohort, the percentage of low secretors in Japan was estimated by measuring the 2′‐FL levels in breast milk. Maternal secretion status is classified into four genotypes according to the variants of *FUT2* (*Sec* [+]/[–]), and *FUT3* (*Le* [+]/[–]) genes (Zhang et al., 2021). Approximately 15–20% of mothers worldwide are classified as *Sec* (–) (Pérez‐Escalante et al., 2022). In this study, 24 of 150 samples (16.0%) were estimated as *Sec* (–) based on breast milk 2′‐FL levels (Figure 3). Some review articles have reported that the ratios of *Sec* (–) mothers vary across geographic regions (Thum et al., 2021; van Leeuwen, 2019); however, there are few reports illustrating the ratios in Japan. Narimatsu et al. (1998) and Kudo et al. (1996) reported that inactivated *FUT2* genes in the Japanese population were mainly *sej* alleles, with a frequency of approximately 40%. These reports indicate that the ratio of the *sej* homozygotes were approximately 16%, agreeing with the ratio of low secretors in this study. Maternal secretor status constitutes a pivotal factor, given its reported association with infant outcomes. Infants fed *Sec* (+) breast milk have an increased number of *Bifidobacteria* in their intestines compared to infants fed *Sec* (–) breast milk (Lewis et al., 2015; Wang et al., 2023). Furthermore, Wang et al. (2023) reported that metabolites in the stool of infants varied according to the maternal secretor status. Given that metabolites from intestinal bacteria are key factors in the association between HMOs and outcomes in children (Wall et al., 2014; Yu et al., 2013), maternal secretor status has an important association with outcomes in children. In this study, some neurodevelopmental scores of secretor infants were higher than that of low secretor infants (Table S2). Some researchers have argued concerning the need to implement point‐of‐care and tried to develop a device at bed side to screen the breast milk 2′‐FL levels (Chung et al., 2022). Predicting maternal secretor status from HMO levels in breast milk is a clinically important technique.

The profile of HMOs in breast milk is related to the activity of enzymes that synthesize HMOs. Breast milk 2′‐FL levels were negatively correlated with 3′‐FL levels (Figure 4), which is consistent with other studies (Austin et al., 2019; Menzel et al., 2021; Ren et al., 2023). Breast milk LSTb and DSLNT levels were also negatively correlated with 2′‐FL levels, as indicated in other studies (Ferreira et al., 2020; Pell et al., 2021; Ren et al., 2023). One possible hypothesis is that a higher FUT2 activity more frequently reacts with these HMOs as substrates, although further studies are warranted to verify this hypothesis. Supporting Information S2 sheds more light on this hypothesis using correlations between 2′‐FL, 3′‐FL, and difucosyllactose levels in breast milk. In sialylated HMOs, several positive correlations were mainly observed among sialylated HMOs with the same binding patterns of sialic acids (α2,3‐sialic acid: 3′‐SL, LSTa, DSLNT; α2,6‐sialic acid: 6′‐SL, LSTb, LSTc, DSLNT). Sialylated HMO profiles are expected to associate with the activity of sialyltransferases (STs), such as ST3 and ST6 families (Harduin‐Lepers et al., 2001). To fully understand the HMO profiles in breast milk, more HMOs should be evaluated since breast milk has been reported to comprise more than 150 HMOs (Tadasu Urashima et al., 2018). However, the limited number of analytical standards makes it difficult to measure a wide variety of HMOs. Understanding the cofluctuation of HMOs in breast milk helps characterize the HMO profile.

HMO quantification in breast milk has yielded findings that certain HMOs are positively associated with HC growth, an important indicator of neurodevelopmental outcomes in infants. Some studies have reported that HC growth and neurodevelopmental scores are closely related (Cheong et al., 2008; Zhu et al., 2022). In the present study, a positive association was also found between ΔHCZ and neurodevelopmental scores in all participants (Table S6). Multiple regression analysis for all participants demonstrated that ΔHCZ was positively associated with LSTb levels (at 0–1 month: β = 3.37, 95% CI = 0.22–6.52) and 2′‐FL levels (at 0–5 months: β = 0.21, 95% CI = 0.02–0.40) (Table 8). Breast milk 2′‐FL levels also showed a positive association with ΔHCZ in secretors (at 0–5 months: β = 0.33, 95% CI = 0.07–0.59; at 0–9 months: β = 0.31, 95% CI = 0.02–0.59) (Table 9). In several animal studies, 2′‐FL has been shown to develop brain tissue and cognitive function (Fleming et al., 2020; Oliveros et al., 2016; Vazquez et al., 2016; Vázquez et al., 2015). In a cohort study by Mansell et al. (2023), 2′‐FL was positively associated with HC growth in low secretors. However, there are few reports on the association between sialylated HMOs and HC growth. Although evidence regarding the function of each HMO needs to be accumulated, the present study provides support for the positive association between some HMOs (2′‐FL and LSTb) and HC growth in infants.

This study supports that the breast milk 2′‐FL levels were positively associated with the child's neurodevelopmental scores in addition to head growth. In an evaluation of ordinal logistic regression analysis for all participants, 2′‐FL levels were positively associated with fine motor skills at 24 months (cOR = 1.41, 95% CI = 1.06–1.88, Table 9). The cohort studies by Berger et al. (2020), Oliveros et al. (2020), and Willemsen et al. (2023) also showed that the 2′‐FL levels were positively associated with a child's neurodevelopmental scores. Although a causal association is required for validation, the present study supports the notion that the 2′‐FL levels are associated with head growth in early life and with subsequent higher neurodevelopmental scores.

The genotype of HMO secretion may be involved in the association between sialylated HMO levels in breast milk and higher neurodevelopmental scores in children. Ordinal logistic regression analysis for all participants showed no positive association between each sialylated HMO level and neurodevelopmental scores, whereas ordinal logistic regression analysis for secretors showed a positive association between the 3′‐SL levels and gross motor skills at 12 months (cOR = 2.39, 95% CI = 1.05–5.80) and between the DSLNT levels and fine motor skills at 12 months (cOR = 1.45, 95% CI = 1.05–2.02) (Table 9). One hypothesis is that the presence of a sufficient amount of particular HMOs, such as 2′‐FL, leads to show the positive association between sialylated HMOs and neurodevelopment in children. However, Oliveros et al. (2020) reported a positive association between breast milk 6′‐SL levels and cognitive scores at 18 months in all participants, which is not consistent with this study. Thus, further studies are warranted to determine whether the association with HMO levels is influenced by genotype. The possible effects of sialylated HMOs are thought to be via the supplementation of sialic acid, which is released by intestinal bacteria (ten Bruggencate et al., 2014). This may be one of the factors to cause inconsistent results across studies since intestinal bacteria vary across different populations (Syromyatnikov et al., 2022).

There are some limitations of the studies for the evaluation of HMOs in breast milk samples from the TMM BirThree cohort. First, the conditions for breast milk collection were not unified, even though breast milk composition can vary considerably due to factors such as fore‐ or hindmilk (Mitoulas et al., 2002) and the time of day (Italianer et al., 2020). This variability arose because the study utilized biobanked breast milk samples that were not specifically collected for this research. During the establishment of the biobank, the primary focus was on maximizing sample collection rather than standardizing collection conditions. As Leghi et al. (2018) suggested, a methodological study to establish a “gold standard” sampling method is necessary. Second, stratification based on the *Le* genotype was not performed because HMOs with α1,4‐fucose were not among the target analytes in this study. Including stratification by the *Le* genotype, in addition to the *Sec* genotype, might provide a deeper understanding of the associations between HMO levels in breast milk and ΔHCZ or ASQ‐3 scores. However, previous reports indicate that the prevalence of *Le* (–) individuals is considerably lower than that of *Sec* (–) individuals (Cho et al., 2021; Ren et al., 2023; Sudarma et al., 2023). Third, this was an observational study, meaning that the causal association between the factors and outcomes is unknown. Although the multivariate analysis was performed to adjust for potentially confounding factors, which may be associated with infants’ neurodevelopmental outcomes, other unknown factors might have influenced the findings of this study.

## 5 CONCLUSION

The developed quantification methods for eight HMOs were used to evaluate the HMO profile in breast milk and estimate the genotype of the maternal secretion status in the Japanese Cohort. Although the statistical models used in the multivariate analysis were exploratory, certain HMO levels obtained by these methods were associated with head growth and higher neurodevelopmental scores, marking the first such report in a Japanese cohort. The quantification methods in this study provided additional insights into the characteristics of HMO profiles in breast milk and their potential associations with infant outcomes.

## AUTHOR CONTRIBUTIONS

**Keigo Sato**: Methodology; writing—original draft; formal analysis; validation; investigation; data curation. **Yoshitaka Nakamura**: Conceptualization; writing—review and editing; resources. **Kazuhito Fujiyama**: Methodology; writing—review and editing. **Kinuko Ohneda**: Conceptualization; funding acquisition; supervision; writing—review and editing. **Takahiro Nobukuni**: Writing—review and editing. **Soichi Ogishima**: Writing—review and editing. **Satoshi Mizuno**: Writing—review and editing. **Seizo Koshiba**: Writing—review and editing. **Shinichi Kuriyama**: Writing—review and editing. **Shinji Jinno**: Writing—review and editing; supervision; investigation; project administration; visualization.

## CONFLICT OF INTEREST STATEMENT

This study was funded by Meiji Co., Ltd. The TMM BirThree Cohort Study was supported in part by the MEXT TMM project and the Japan Agency of Medical Research Development (grant numbers JP21tm0124005, JP21tm0124005, and JP21tm0424601). K. S., Y. N., and S. J. used to be employees of Meiji Co., Ltd. K. S. and S. J. are employees of Meiji Holdings Co., Ltd.

## Supporting information



Supplementary Materials.
