# Supplementary material for: Absolute quantification of eight human milk oligosaccharides in breast milk to evaluate their concentration profiles and associations with infants’ neurodevelopmental outcomes
Source: J Food Sci. 2024 Dec 10;89(12):10152–70. doi: 10.1111/1750-3841.17597 (PMC11673463; doi:10.1111/1750-3841.17597)
Supplement: Supplementary file 1 — Supplementary Materials. [file JFDS-89-10152-s001.pptx]

## Slide 1
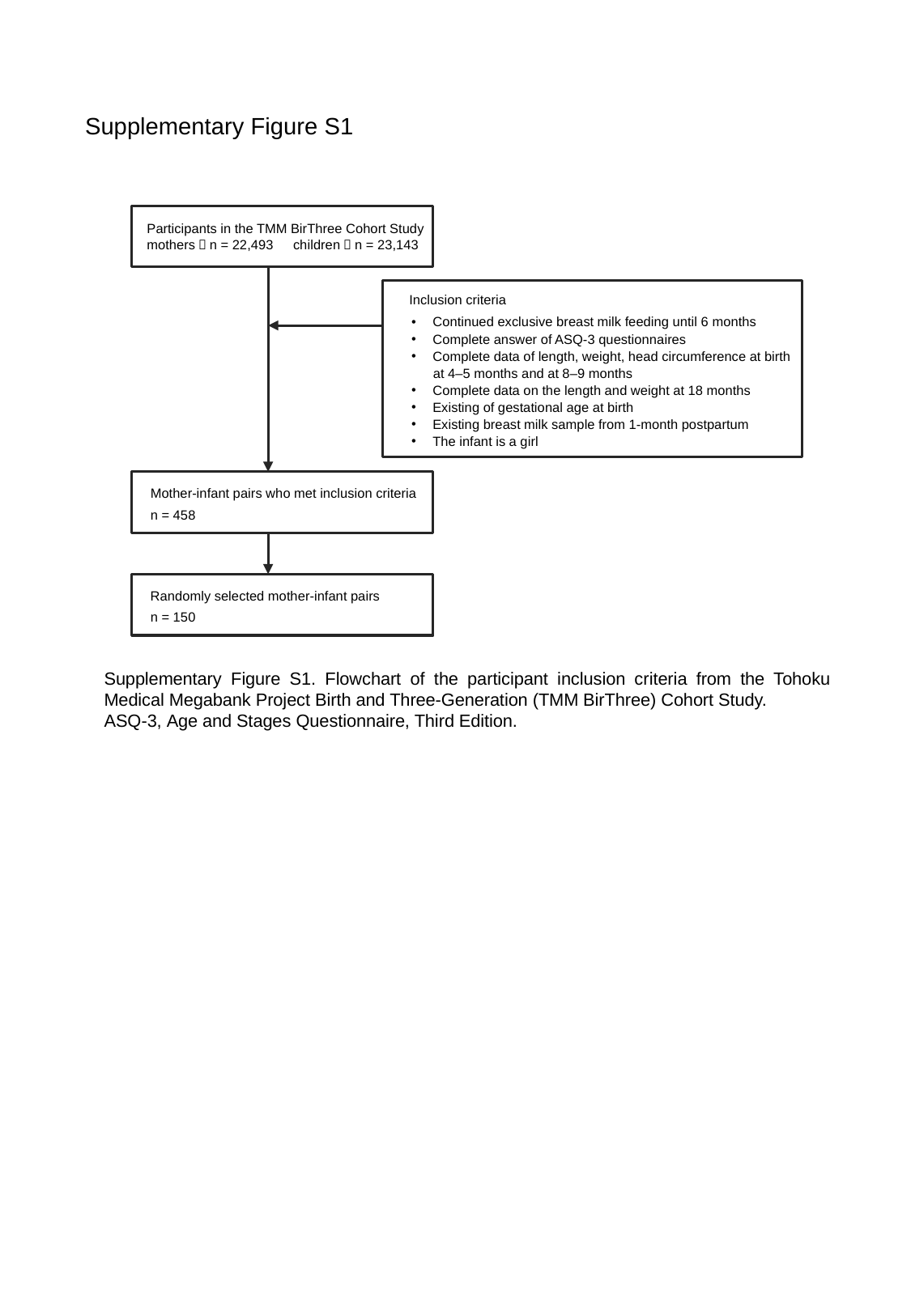

Supplementary Figure S1
Participants in the TMM BirThree Cohort Study
mothers：n = 22,493　children：n = 23,143
Inclusion criteria
Continued exclusive breast milk feeding until 6 months
Complete answer of ASQ-3 questionnaires
Complete data of length, weight, head circumference at birth
 at 4–5 months and at 8–9 months
Complete data on the length and weight at 18 months
Existing of gestational age at birth
Existing breast milk sample from 1-month postpartum
The infant is a girl
Mother-infant pairs who met inclusion criteria
n = 458
Randomly selected mother-infant pairs
n = 150
Supplementary Figure S1. Flowchart of the participant inclusion criteria from the Tohoku Medical Megabank Project Birth and Three-Generation (TMM BirThree) Cohort Study.
ASQ-3, Age and Stages Questionnaire, Third Edition.

## Slide 2
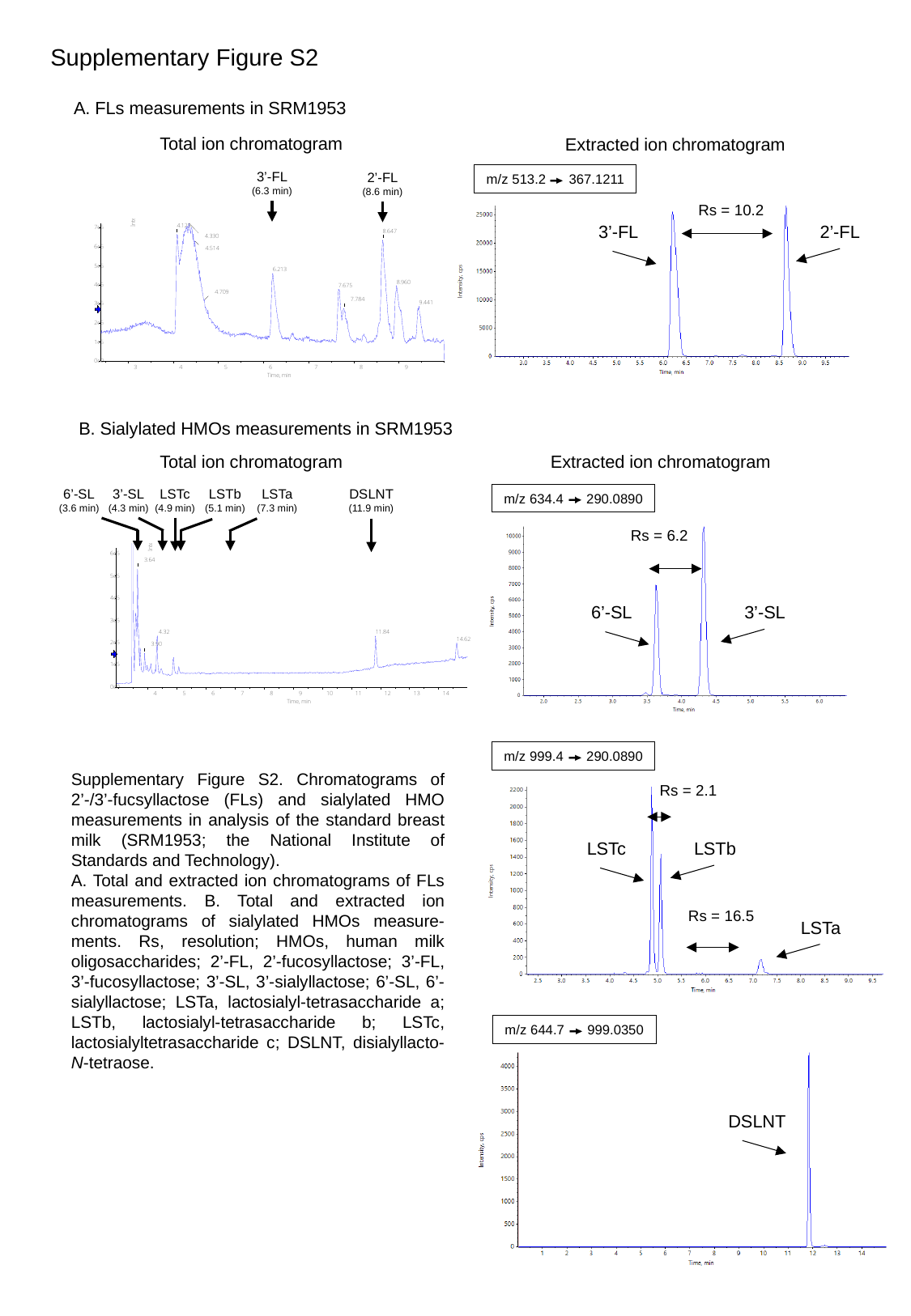

Supplementary Figure S2
A. FLs measurements in SRM1953
Total ion chromatogram
Extracted ion chromatogram
3’-FL
(6.3 min)
2’-FL
(8.6 min)
m/z 513.2 367.1211
Rs = 10.2
3’-FL
2’-FL
B. Sialylated HMOs measurements in SRM1953
Extracted ion chromatogram
Total ion chromatogram
6’-SL
(3.6 min)
3’-SL
(4.3 min)
LSTc
(4.9 min)
LSTb
(5.1 min)
LSTa
(7.3 min)
DSLNT
(11.9 min)
m/z 634.4 290.0890
Rs = 6.2
6’-SL
3’-SL
m/z 999.4 290.0890
Supplementary Figure S2. Chromatograms of 2’-/3’-fucsyllactose (FLs) and sialylated HMO measurements in analysis of the standard breast milk (SRM1953; the National Institute of Standards and Technology).
A. Total and extracted ion chromatograms of FLs measurements. B. Total and extracted ion chromatograms of sialylated HMOs measure-ments. Rs, resolution; HMOs, human milk oligosaccharides; 2’-FL, 2’-fucosyllactose; 3’-FL, 3’-fucosyllactose; 3’-SL, 3’-sialyllactose; 6’-SL, 6’-sialyllactose; LSTa, lactosialyl-tetrasaccharide a; LSTb, lactosialyl-tetrasaccharide b; LSTc, lactosialyltetrasaccharide c; DSLNT, disialyllacto-N-tetraose.
Rs = 2.1
LSTc
LSTb
Rs = 16.5
LSTa
m/z 644.7 999.0350
DSLNT

## Slide 3
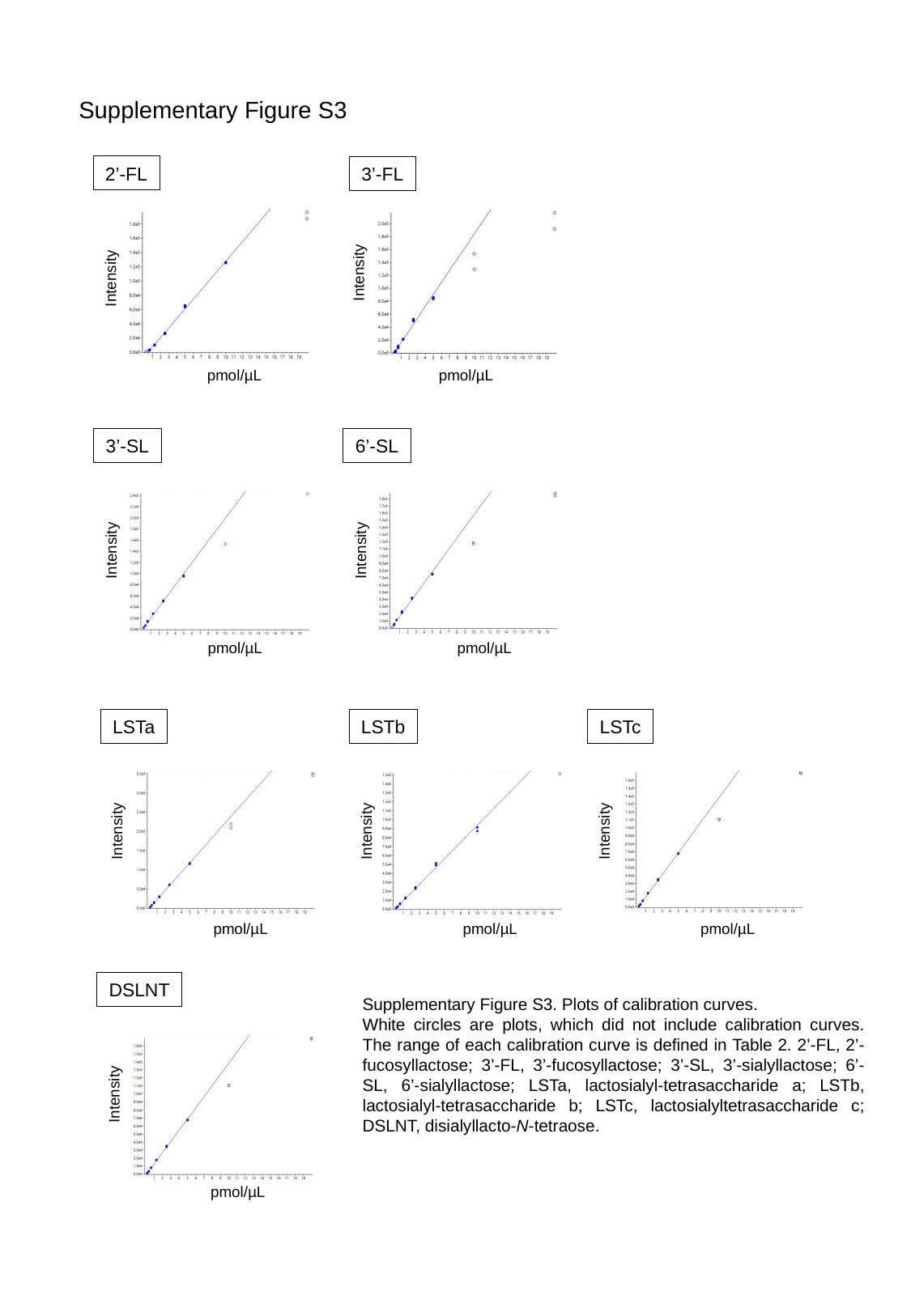

Supplementary Figure S3
2’-FL
3’-FL
Intensity
Intensity
pmol/µL
pmol/µL
3’-SL
6’-SL
Intensity
Intensity
pmol/µL
pmol/µL
LSTa
LSTb
LSTc
Intensity
Intensity
Intensity
pmol/µL
pmol/µL
pmol/µL
DSLNT
Supplementary Figure S3. Plots of calibration curves.
White circles are plots, which did not include calibration curves. The range of each calibration curve is defined in Table 2. 2’-FL, 2’-fucosyllactose; 3’-FL, 3’-fucosyllactose; 3’-SL, 3’-sialyllactose; 6’-SL, 6’-sialyllactose; LSTa, lactosialyl-tetrasaccharide a; LSTb, lactosialyl-tetrasaccharide b; LSTc, lactosialyltetrasaccharide c; DSLNT, disialyllacto-N-tetraose.
Intensity
pmol/µL

## Slide 4
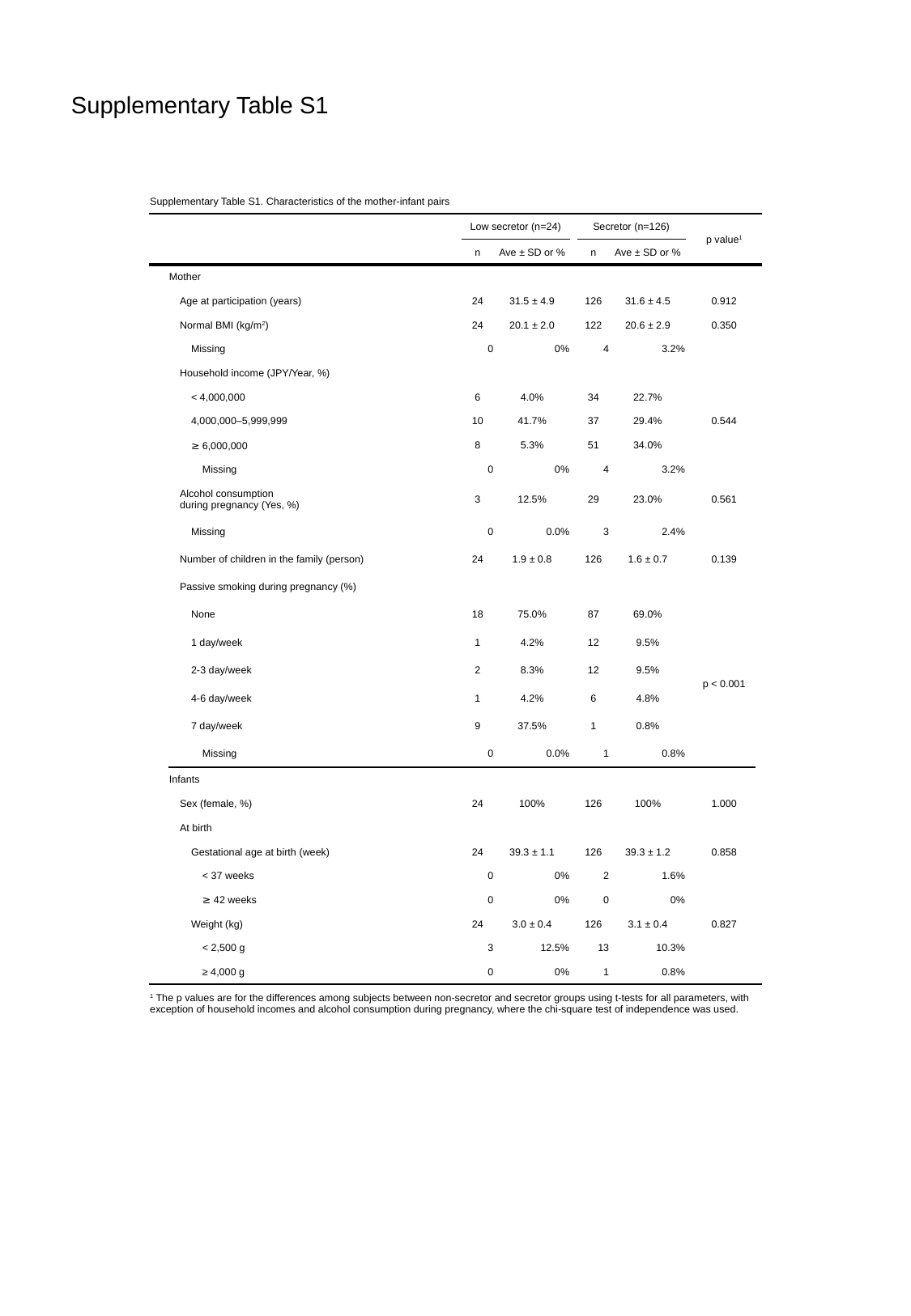

Supplementary Table S1
| Supplementary Table S1. Characteristics of the mother-infant pairs | | | | | | | | | |
| --- | --- | --- | --- | --- | --- | --- | --- | --- | --- |
| | | Low secretor (n=24) | | | Secretor (n=126) | | | p value1 | |
| | | n | Ave ± SD or % | | n | Ave ± SD or % | | | |
| | Mother | | | | | | | | |
| | Age at participation (years) | 24 | 31.5 ± 4.9 | | 126 | 31.6 ± 4.5 | | 0.912 | |
| | Normal BMI (kg/m2) | 24 | 20.1 ± 2.0 | | 122 | 20.6 ± 2.9 | | 0.350 | |
| | Missing | 0 | 0% | | 4 | 3.2% | | | |
| | Household income (JPY/Year, %) | | | | | | | | |
| | < 4,000,000 | 6 | 4.0% | | 34 | 22.7% | | 0.544 | |
| | 4,000,000–5,999,999 | 10 | 41.7% | | 37 | 29.4% | | | |
| | ≥ 6,000,000 | 8 | 5.3% | | 51 | 34.0% | | | |
| | Missing | 0 | 0% | | 4 | 3.2% | | | |
| | Alcohol consumption during pregnancy (Yes, %) | 3 | 12.5% | | 29 | 23.0% | | 0.561 | |
| | Missing | 0 | 0.0% | | 3 | 2.4% | | | |
| | Number of children in the family (person) | 24 | 1.9 ± 0.8 | | 126 | 1.6 ± 0.7 | | 0.139 | |
| | Passive smoking during pregnancy (%) | | | | | | | | |
| | None | 18 | 75.0% | | 87 | 69.0% | | p < 0.001 | |
| | 1 day/week | 1 | 4.2% | | 12 | 9.5% | | | |
| | 2-3 day/week | 2 | 8.3% | | 12 | 9.5% | | | |
| | 4-6 day/week | 1 | 4.2% | | 6 | 4.8% | | | |
| | 7 day/week | 9 | 37.5% | | 1 | 0.8% | | | |
| | Missing | 0 | 0.0% | | 1 | 0.8% | | | |
| | Infants | | | | | | | | |
| | Sex (female, %) | 24 | 100% | | 126 | 100% | | 1.000 | |
| | At birth | | | | | | | | |
| | Gestational age at birth (week) | 24 | 39.3 ± 1.1 | | 126 | 39.3 ± 1.2 | | 0.858 | |
| | < 37 weeks | 0 | 0% | | 2 | 1.6% | | | |
| | ≥ 42 weeks | 0 | 0% | | 0 | 0% | | | |
| | Weight (kg) | 24 | 3.0 ± 0.4 | | 126 | 3.1 ± 0.4 | | 0.827 | |
| | < 2,500 g | 3 | 12.5% | | 13 | 10.3% | | | |
| | ≥ 4,000 g | 0 | 0% | | 1 | 0.8% | | | |
| 1 The p values are for the differences among subjects between non-secretor and secretor groups using t-tests for all parameters, with exception of household incomes and alcohol consumption during pregnancy, where the chi-square test of independence was used. | | | | | | | | | |

## Slide 5
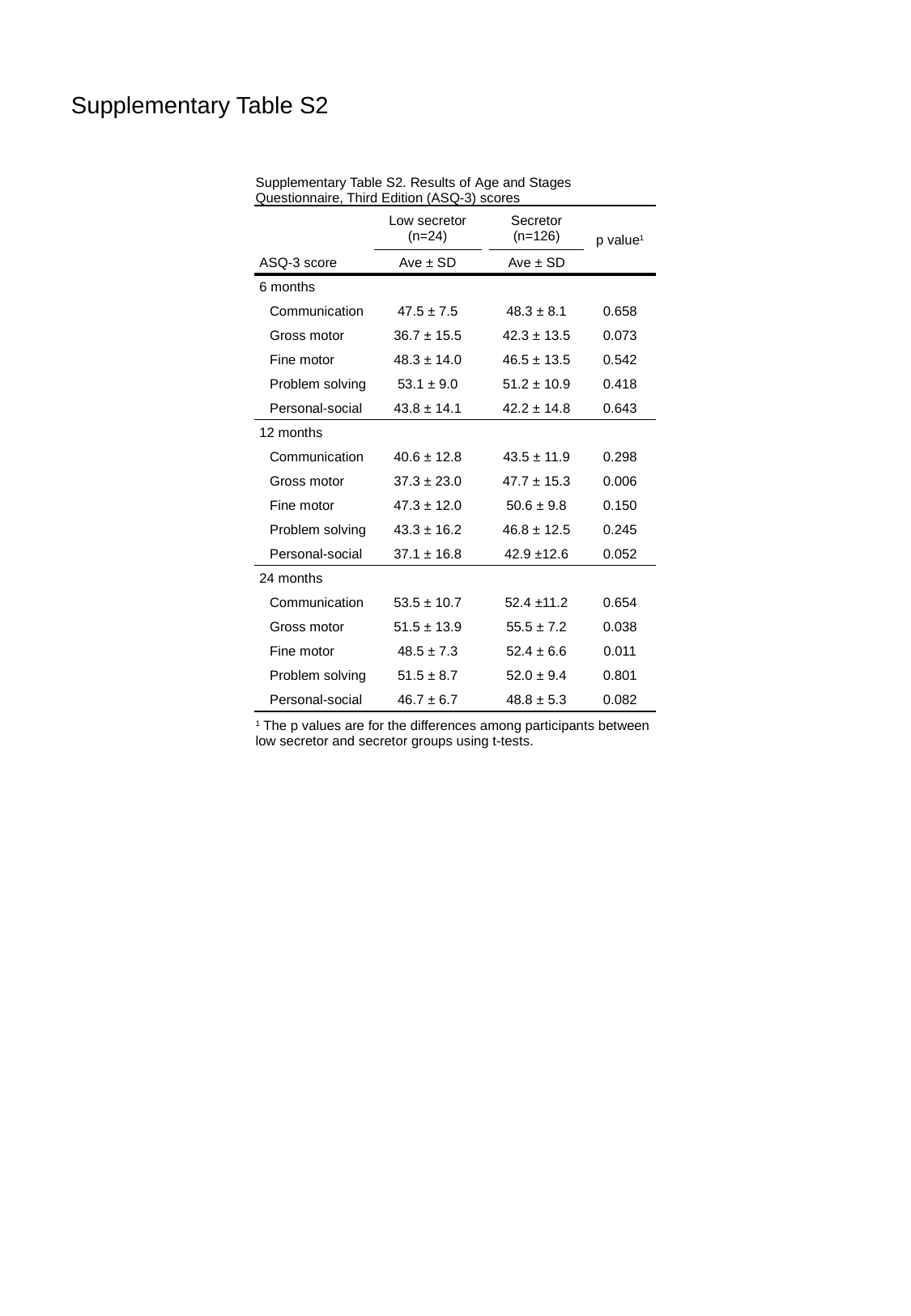

Supplementary Table S2
| Supplementary Table S2. Results of Age and Stages Questionnaire, Third Edition (ASQ-3) scores | | | | | | |
| --- | --- | --- | --- | --- | --- | --- |
| | Low secretor (n=24) | | Secretor (n=126) | | p value1 | |
| ASQ-3 score | Ave ± SD | | Ave ± SD | | | |
| 6 months | | | | | | |
| Communication | 47.5 ± 7.5 | | 48.3 ± 8.1 | | 0.658 | |
| Gross motor | 36.7 ± 15.5 | | 42.3 ± 13.5 | | 0.073 | |
| Fine motor | 48.3 ± 14.0 | | 46.5 ± 13.5 | | 0.542 | |
| Problem solving | 53.1 ± 9.0 | | 51.2 ± 10.9 | | 0.418 | |
| Personal-social | 43.8 ± 14.1 | | 42.2 ± 14.8 | | 0.643 | |
| 12 months | | | | | | |
| Communication | 40.6 ± 12.8 | | 43.5 ± 11.9 | | 0.298 | |
| Gross motor | 37.3 ± 23.0 | | 47.7 ± 15.3 | | 0.006 | |
| Fine motor | 47.3 ± 12.0 | | 50.6 ± 9.8 | | 0.150 | |
| Problem solving | 43.3 ± 16.2 | | 46.8 ± 12.5 | | 0.245 | |
| Personal-social | 37.1 ± 16.8 | | 42.9 ±12.6 | | 0.052 | |
| 24 months | | | | | | |
| Communication | 53.5 ± 10.7 | | 52.4 ±11.2 | | 0.654 | |
| Gross motor | 51.5 ± 13.9 | | 55.5 ± 7.2 | | 0.038 | |
| Fine motor | 48.5 ± 7.3 | | 52.4 ± 6.6 | | 0.011 | |
| Problem solving | 51.5 ± 8.7 | | 52.0 ± 9.4 | | 0.801 | |
| Personal-social | 46.7 ± 6.7 | | 48.8 ± 5.3 | | 0.082 | |
| 1 The p values are for the differences among participants between low secretor and secretor groups using t-tests. | | | | | | |

## Slide 6
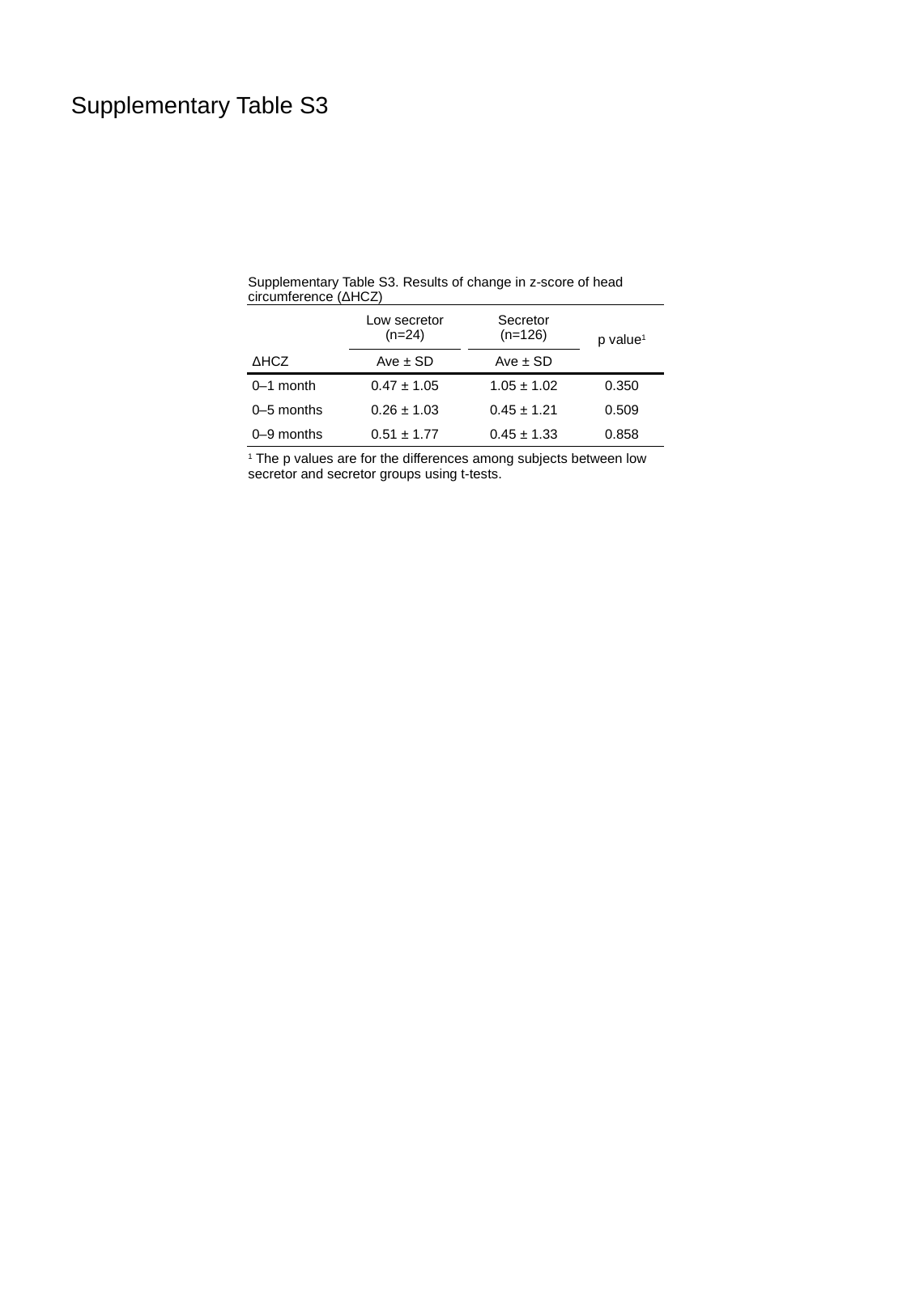

Supplementary Table S3
| Supplementary Table S3. Results of change in z-score of head circumference (ΔHCZ) | | | | | | |
| --- | --- | --- | --- | --- | --- | --- |
| | Low secretor (n=24) | | Secretor (n=126) | | p value1 | |
| ΔHCZ | Ave ± SD | | Ave ± SD | | | |
| 0–1 month | 0.47 ± 1.05 | | 1.05 ± 1.02 | | 0.350 | |
| 0–5 months | 0.26 ± 1.03 | | 0.45 ± 1.21 | | 0.509 | |
| 0–9 months | 0.51 ± 1.77 | | 0.45 ± 1.33 | | 0.858 | |
| 1 The p values are for the differences among subjects between low secretor and secretor groups using t-tests. | | | | | | |

## Slide 7
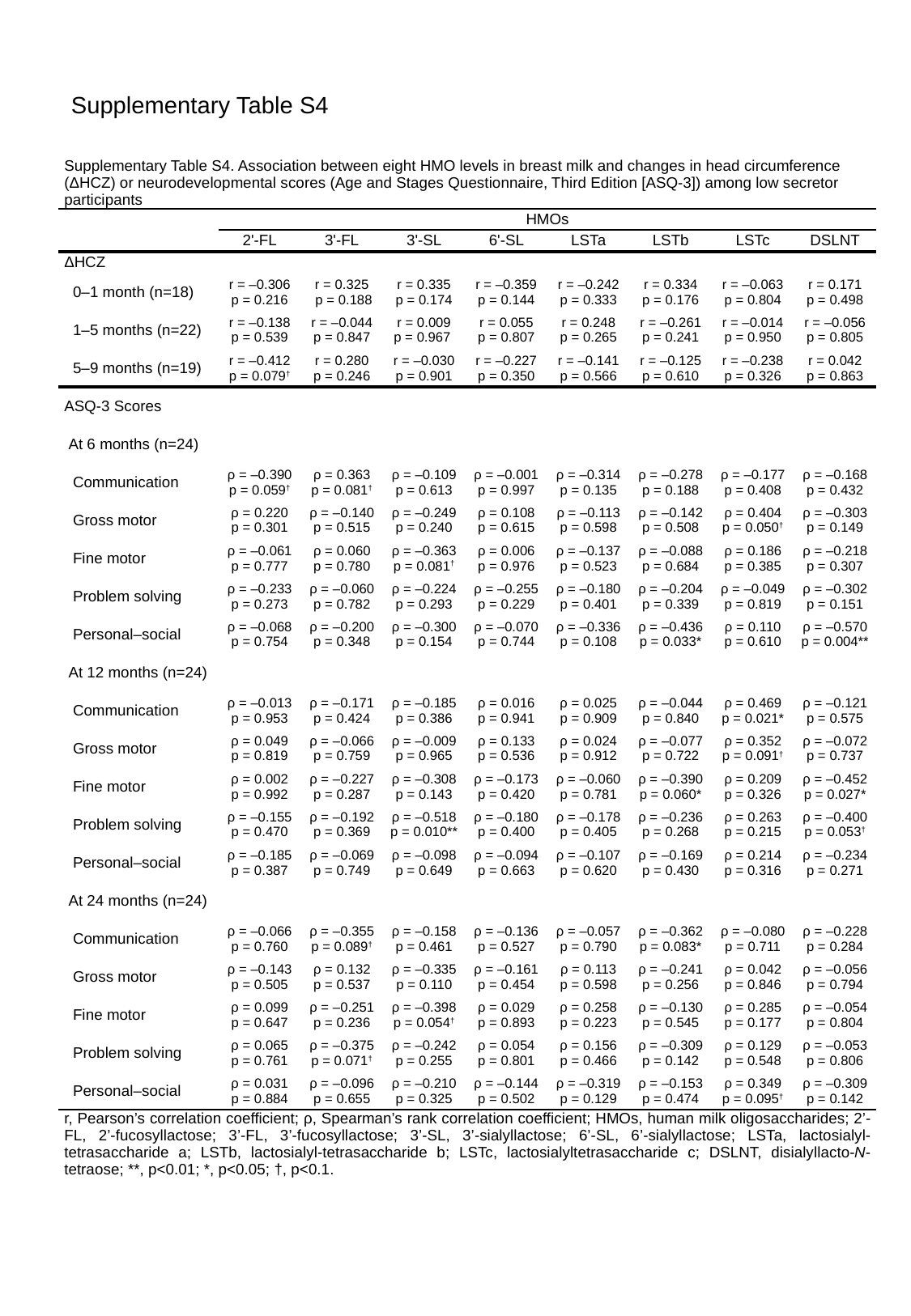

Supplementary Table S4
| Supplementary Table S4. Association between eight HMO levels in breast milk and changes in head circumference (ΔHCZ) or neurodevelopmental scores (Age and Stages Questionnaire, Third Edition [ASQ-3]) among low secretor participants | | | | | | | | |
| --- | --- | --- | --- | --- | --- | --- | --- | --- |
| | HMOs | | | | | | | |
| | 2'-FL | 3'-FL | 3'-SL | 6'-SL | LSTa | LSTb | LSTc | DSLNT |
| ΔHCZ | | | | | | | | |
| 0–1 month (n=18) | r = –0.306 p = 0.216 | r = 0.325 p = 0.188 | r = 0.335 p = 0.174 | r = –0.359 p = 0.144 | r = –0.242 p = 0.333 | r = 0.334 p = 0.176 | r = –0.063 p = 0.804 | r = 0.171 p = 0.498 |
| 1–5 months (n=22) | r = –0.138 p = 0.539 | r = –0.044 p = 0.847 | r = 0.009 p = 0.967 | r = 0.055 p = 0.807 | r = 0.248 p = 0.265 | r = –0.261 p = 0.241 | r = –0.014 p = 0.950 | r = –0.056 p = 0.805 |
| 5–9 months (n=19) | r = –0.412 p = 0.079† | r = 0.280 p = 0.246 | r = –0.030 p = 0.901 | r = –0.227 p = 0.350 | r = –0.141 p = 0.566 | r = –0.125 p = 0.610 | r = –0.238 p = 0.326 | r = 0.042 p = 0.863 |
| ASQ-3 Scores | | | | | | | | |
| At 6 months (n=24) | | | | | | | | |
| Communication | ρ = –0.390 p = 0.059† | ρ = 0.363 p = 0.081† | ρ = –0.109 p = 0.613 | ρ = –0.001 p = 0.997 | ρ = –0.314 p = 0.135 | ρ = –0.278 p = 0.188 | ρ = –0.177 p = 0.408 | ρ = –0.168 p = 0.432 |
| Gross motor | ρ = 0.220 p = 0.301 | ρ = –0.140 p = 0.515 | ρ = –0.249 p = 0.240 | ρ = 0.108 p = 0.615 | ρ = –0.113 p = 0.598 | ρ = –0.142 p = 0.508 | ρ = 0.404 p = 0.050† | ρ = –0.303 p = 0.149 |
| Fine motor | ρ = –0.061 p = 0.777 | ρ = 0.060 p = 0.780 | ρ = –0.363 p = 0.081† | ρ = 0.006 p = 0.976 | ρ = –0.137 p = 0.523 | ρ = –0.088 p = 0.684 | ρ = 0.186 p = 0.385 | ρ = –0.218 p = 0.307 |
| Problem solving | ρ = –0.233 p = 0.273 | ρ = –0.060 p = 0.782 | ρ = –0.224 p = 0.293 | ρ = –0.255 p = 0.229 | ρ = –0.180 p = 0.401 | ρ = –0.204 p = 0.339 | ρ = –0.049 p = 0.819 | ρ = –0.302 p = 0.151 |
| Personal–social | ρ = –0.068 p = 0.754 | ρ = –0.200 p = 0.348 | ρ = –0.300 p = 0.154 | ρ = –0.070 p = 0.744 | ρ = –0.336 p = 0.108 | ρ = –0.436 p = 0.033\* | ρ = 0.110 p = 0.610 | ρ = –0.570 p = 0.004\*\* |
| At 12 months (n=24) | | | | | | | | |
| Communication | ρ = –0.013 p = 0.953 | ρ = –0.171 p = 0.424 | ρ = –0.185 p = 0.386 | ρ = 0.016 p = 0.941 | ρ = 0.025 p = 0.909 | ρ = –0.044 p = 0.840 | ρ = 0.469 p = 0.021\* | ρ = –0.121 p = 0.575 |
| Gross motor | ρ = 0.049 p = 0.819 | ρ = –0.066 p = 0.759 | ρ = –0.009 p = 0.965 | ρ = 0.133 p = 0.536 | ρ = 0.024 p = 0.912 | ρ = –0.077 p = 0.722 | ρ = 0.352 p = 0.091† | ρ = –0.072 p = 0.737 |
| Fine motor | ρ = 0.002 p = 0.992 | ρ = –0.227 p = 0.287 | ρ = –0.308 p = 0.143 | ρ = –0.173 p = 0.420 | ρ = –0.060 p = 0.781 | ρ = –0.390 p = 0.060\* | ρ = 0.209 p = 0.326 | ρ = –0.452 p = 0.027\* |
| Problem solving | ρ = –0.155 p = 0.470 | ρ = –0.192 p = 0.369 | ρ = –0.518 p = 0.010\*\* | ρ = –0.180 p = 0.400 | ρ = –0.178 p = 0.405 | ρ = –0.236 p = 0.268 | ρ = 0.263 p = 0.215 | ρ = –0.400 p = 0.053† |
| Personal–social | ρ = –0.185 p = 0.387 | ρ = –0.069 p = 0.749 | ρ = –0.098 p = 0.649 | ρ = –0.094 p = 0.663 | ρ = –0.107 p = 0.620 | ρ = –0.169 p = 0.430 | ρ = 0.214 p = 0.316 | ρ = –0.234 p = 0.271 |
| At 24 months (n=24) | | | | | | | | |
| Communication | ρ = –0.066 p = 0.760 | ρ = –0.355 p = 0.089† | ρ = –0.158 p = 0.461 | ρ = –0.136 p = 0.527 | ρ = –0.057 p = 0.790 | ρ = –0.362 p = 0.083\* | ρ = –0.080 p = 0.711 | ρ = –0.228 p = 0.284 |
| Gross motor | ρ = –0.143 p = 0.505 | ρ = 0.132 p = 0.537 | ρ = –0.335 p = 0.110 | ρ = –0.161 p = 0.454 | ρ = 0.113 p = 0.598 | ρ = –0.241 p = 0.256 | ρ = 0.042 p = 0.846 | ρ = –0.056 p = 0.794 |
| Fine motor | ρ = 0.099 p = 0.647 | ρ = –0.251 p = 0.236 | ρ = –0.398 p = 0.054† | ρ = 0.029 p = 0.893 | ρ = 0.258 p = 0.223 | ρ = –0.130 p = 0.545 | ρ = 0.285 p = 0.177 | ρ = –0.054 p = 0.804 |
| Problem solving | ρ = 0.065 p = 0.761 | ρ = –0.375 p = 0.071† | ρ = –0.242 p = 0.255 | ρ = 0.054 p = 0.801 | ρ = 0.156 p = 0.466 | ρ = –0.309 p = 0.142 | ρ = 0.129 p = 0.548 | ρ = –0.053 p = 0.806 |
| Personal–social | ρ = 0.031 p = 0.884 | ρ = –0.096 p = 0.655 | ρ = –0.210 p = 0.325 | ρ = –0.144 p = 0.502 | ρ = –0.319 p = 0.129 | ρ = –0.153 p = 0.474 | ρ = 0.349 p = 0.095† | ρ = –0.309 p = 0.142 |
| r, Pearson’s correlation coefficient; ρ, Spearman’s rank correlation coefficient; HMOs, human milk oligosaccharides; 2’-FL, 2’-fucosyllactose; 3’-FL, 3’-fucosyllactose; 3’-SL, 3’-sialyllactose; 6’-SL, 6’-sialyllactose; LSTa, lactosialyl-tetrasaccharide a; LSTb, lactosialyl-tetrasaccharide b; LSTc, lactosialyltetrasaccharide c; DSLNT, disialyllacto-N-tetraose; \*\*, p<0.01; \*, p<0.05; †, p<0.1. | | | | | | | | |

## Slide 8
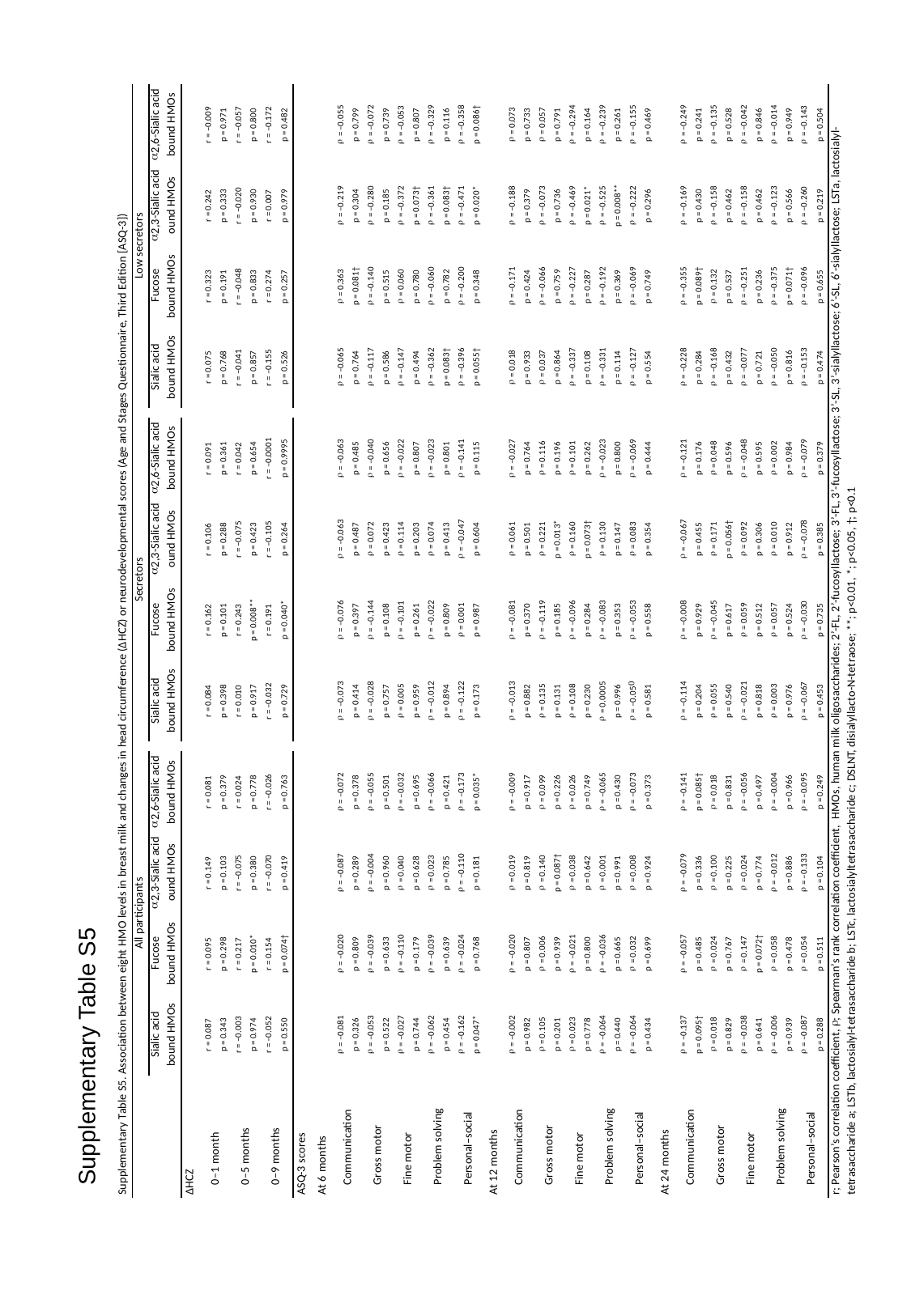

Supplementary Table S5

## Slide 9
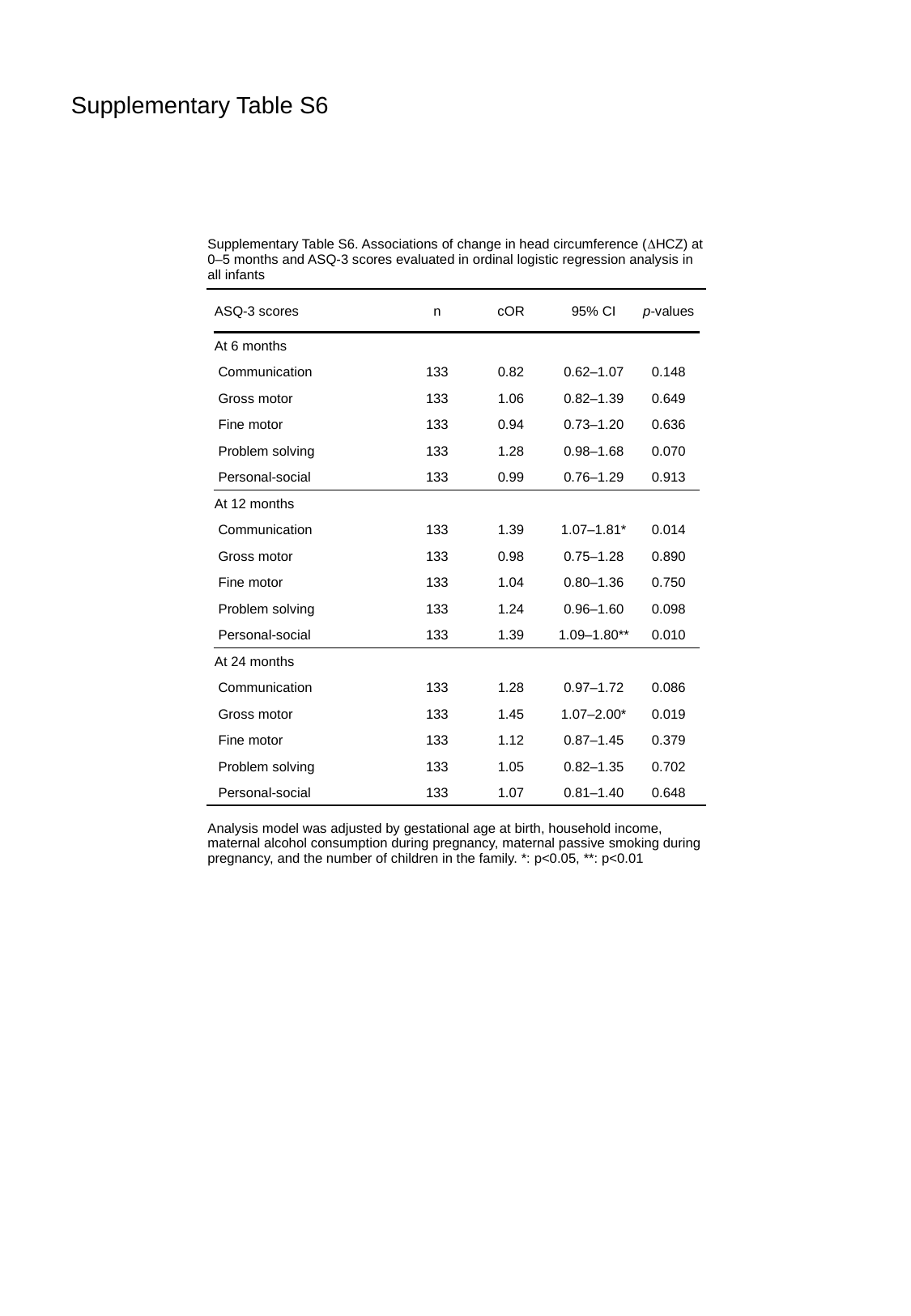

Supplementary Table S6
| Supplementary Table S6. Associations of change in head circumference (DHCZ) at 0–5 months and ASQ-3 scores evaluated in ordinal logistic regression analysis in all infants | | | | | | |
| --- | --- | --- | --- | --- | --- | --- |
| | ASQ-3 scores | n | cOR | 95% CI | p-values | |
| | At 6 months | | | | | |
| | Communication | 133 | 0.82 | 0.62–1.07 | 0.148 | |
| | Gross motor | 133 | 1.06 | 0.82–1.39 | 0.649 | |
| | Fine motor | 133 | 0.94 | 0.73–1.20 | 0.636 | |
| | Problem solving | 133 | 1.28 | 0.98–1.68 | 0.070 | |
| | Personal-social | 133 | 0.99 | 0.76–1.29 | 0.913 | |
| | At 12 months | | | | | |
| | Communication | 133 | 1.39 | 1.07–1.81\* | 0.014 | |
| | Gross motor | 133 | 0.98 | 0.75–1.28 | 0.890 | |
| | Fine motor | 133 | 1.04 | 0.80–1.36 | 0.750 | |
| | Problem solving | 133 | 1.24 | 0.96–1.60 | 0.098 | |
| | Personal-social | 133 | 1.39 | 1.09–1.80\*\* | 0.010 | |
| | At 24 months | | | | | |
| | Communication | 133 | 1.28 | 0.97–1.72 | 0.086 | |
| | Gross motor | 133 | 1.45 | 1.07–2.00\* | 0.019 | |
| | Fine motor | 133 | 1.12 | 0.87–1.45 | 0.379 | |
| | Problem solving | 133 | 1.05 | 0.82–1.35 | 0.702 | |
| | Personal-social | 133 | 1.07 | 0.81–1.40 | 0.648 | |
| Analysis model was adjusted by gestational age at birth, household income, maternal alcohol consumption during pregnancy, maternal passive smoking during pregnancy, and the number of children in the family. \*: p<0.05, \*\*: p<0.01 | | | | | | |

## Slide 10
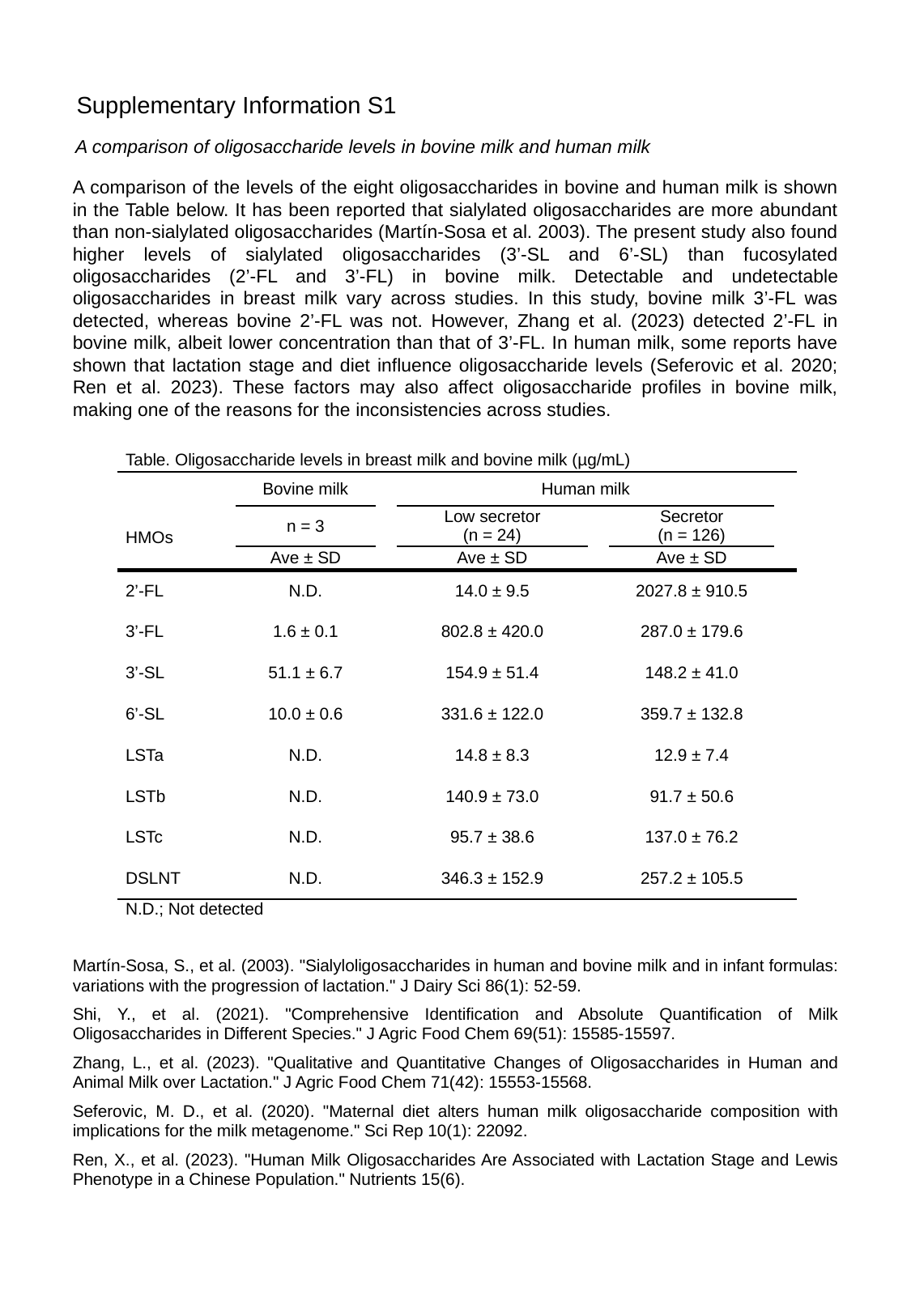

Supplementary Information S1
A comparison of oligosaccharide levels in bovine milk and human milk
A comparison of the levels of the eight oligosaccharides in bovine and human milk is shown in the Table below. It has been reported that sialylated oligosaccharides are more abundant than non-sialylated oligosaccharides (Martín-Sosa et al. 2003). The present study also found higher levels of sialylated oligosaccharides (3’-SL and 6’-SL) than fucosylated oligosaccharides (2’-FL and 3’-FL) in bovine milk. Detectable and undetectable oligosaccharides in breast milk vary across studies. In this study, bovine milk 3’-FL was detected, whereas bovine 2’-FL was not. However, Zhang et al. (2023) detected 2’-FL in bovine milk, albeit lower concentration than that of 3’-FL. In human milk, some reports have shown that lactation stage and diet influence oligosaccharide levels (Seferovic et al. 2020; Ren et al. 2023). These factors may also affect oligosaccharide profiles in bovine milk, making one of the reasons for the inconsistencies across studies.
| Table. Oligosaccharide levels in breast milk and bovine milk (µg/mL) | | | | | | |
| --- | --- | --- | --- | --- | --- | --- |
| | Bovine milk | | Human milk | | | |
| HMOs | n = 3 | | Low secretor (n = 24) | | Secretor (n = 126) | |
| | Ave ± SD | | Ave ± SD | | Ave ± SD | |
| 2’-FL | N.D. | | 14.0 ± 9.5 | | 2027.8 ± 910.5 | |
| 3’-FL | 1.6 ± 0.1 | | 802.8 ± 420.0 | | 287.0 ± 179.6 | |
| 3’-SL | 51.1 ± 6.7 | | 154.9 ± 51.4 | | 148.2 ± 41.0 | |
| 6’-SL | 10.0 ± 0.6 | | 331.6 ± 122.0 | | 359.7 ± 132.8 | |
| LSTa | N.D. | | 14.8 ± 8.3 | | 12.9 ± 7.4 | |
| LSTb | N.D. | | 140.9 ± 73.0 | | 91.7 ± 50.6 | |
| LSTc | N.D. | | 95.7 ± 38.6 | | 137.0 ± 76.2 | |
| DSLNT | N.D. | | 346.3 ± 152.9 | | 257.2 ± 105.5 | |
| N.D.; Not detected | | | | | | |
Martín-Sosa, S., et al. (2003). "Sialyloligosaccharides in human and bovine milk and in infant formulas: variations with the progression of lactation." J Dairy Sci 86(1): 52-59.
Shi, Y., et al. (2021). "Comprehensive Identification and Absolute Quantification of Milk Oligosaccharides in Different Species." J Agric Food Chem 69(51): 15585-15597.
Zhang, L., et al. (2023). "Qualitative and Quantitative Changes of Oligosaccharides in Human and Animal Milk over Lactation." J Agric Food Chem 71(42): 15553-15568.
Seferovic, M. D., et al. (2020). "Maternal diet alters human milk oligosaccharide composition with implications for the milk metagenome." Sci Rep 10(1): 22092.
Ren, X., et al. (2023). "Human Milk Oligosaccharides Are Associated with Lactation Stage and Lewis Phenotype in a Chinese Population." Nutrients 15(6).

## Slide 11
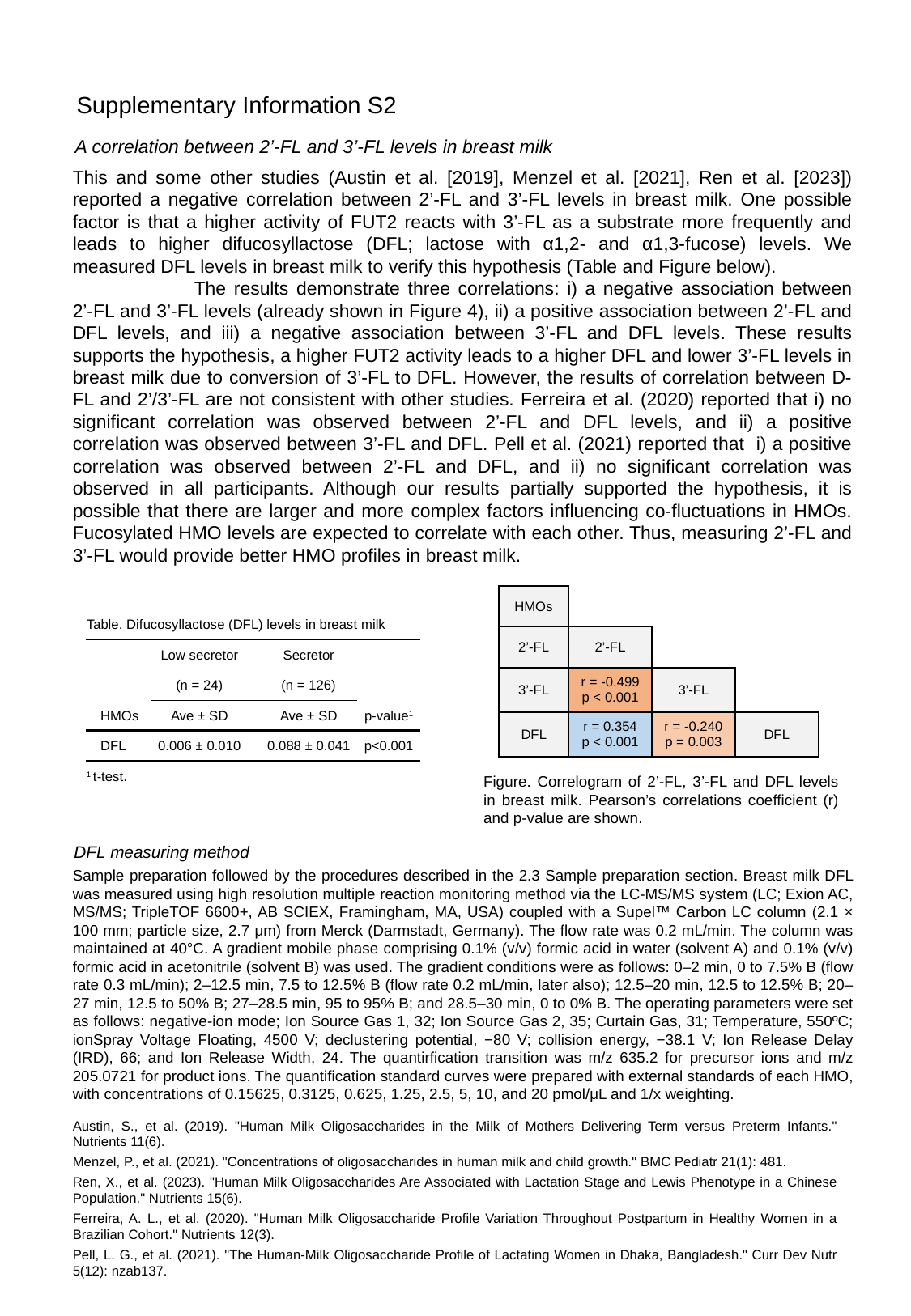

Supplementary Information S2
A correlation between 2’-FL and 3’-FL levels in breast milk
This and some other studies (Austin et al. [2019], Menzel et al. [2021], Ren et al. [2023]) reported a negative correlation between 2’-FL and 3’-FL levels in breast milk. One possible factor is that a higher activity of FUT2 reacts with 3’-FL as a substrate more frequently and leads to higher difucosyllactose (DFL; lactose with α1,2- and α1,3-fucose) levels. We measured DFL levels in breast milk to verify this hypothesis (Table and Figure below).
	The results demonstrate three correlations: i) a negative association between 2’-FL and 3’-FL levels (already shown in Figure 4), ii) a positive association between 2’-FL and DFL levels, and iii) a negative association between 3’-FL and DFL levels. These results supports the hypothesis, a higher FUT2 activity leads to a higher DFL and lower 3’-FL levels in breast milk due to conversion of 3’-FL to DFL. However, the results of correlation between D-FL and 2’/3’-FL are not consistent with other studies. Ferreira et al. (2020) reported that i) no significant correlation was observed between 2’-FL and DFL levels, and ii) a positive correlation was observed between 3’-FL and DFL. Pell et al. (2021) reported that i) a positive correlation was observed between 2’-FL and DFL, and ii) no significant correlation was observed in all participants. Although our results partially supported the hypothesis, it is possible that there are larger and more complex factors influencing co-fluctuations in HMOs. Fucosylated HMO levels are expected to correlate with each other. Thus, measuring 2’-FL and 3’-FL would provide better HMO profiles in breast milk.
| HMOs | | | |
| --- | --- | --- | --- |
| 2’-FL | 2’-FL | | |
| 3’-FL | r = -0.499 p < 0.001 | 3’-FL | |
| DFL | r = 0.354 p < 0.001 | r = -0.240 p = 0.003 | DFL |
| Table. Difucosyllactose (DFL) levels in breast milk | | | | |
| --- | --- | --- | --- | --- |
| | Low secretor | | Secretor | |
| | (n = 24) | | (n = 126) | |
| HMOs | Ave ± SD | | Ave ± SD | p-value1 |
| DFL | 0.006 ± 0.010 | | 0.088 ± 0.041 | p<0.001 |
| 1 t-test. | | | | |
Figure. Correlogram of 2’-FL, 3’-FL and DFL levels in breast milk. Pearson’s correlations coefficient (r) and p-value are shown.
DFL measuring method
Sample preparation followed by the procedures described in the 2.3 Sample preparation section. Breast milk DFL was measured using high resolution multiple reaction monitoring method via the LC-MS/MS system (LC; Exion AC, MS/MS; TripleTOF 6600+, AB SCIEX, Framingham, MA, USA) coupled with a Supel™ Carbon LC column (2.1 × 100 mm; particle size, 2.7 μm) from Merck (Darmstadt, Germany). The flow rate was 0.2 mL/min. The column was maintained at 40°C. A gradient mobile phase comprising 0.1% (v/v) formic acid in water (solvent A) and 0.1% (v/v) formic acid in acetonitrile (solvent B) was used. The gradient conditions were as follows: 0–2 min, 0 to 7.5% B (flow rate 0.3 mL/min); 2–12.5 min, 7.5 to 12.5% B (flow rate 0.2 mL/min, later also); 12.5–20 min, 12.5 to 12.5% B; 20–27 min, 12.5 to 50% B; 27–28.5 min, 95 to 95% B; and 28.5–30 min, 0 to 0% B. The operating parameters were set as follows: negative-ion mode; Ion Source Gas 1, 32; Ion Source Gas 2, 35; Curtain Gas, 31; Temperature, 550ºC; ionSpray Voltage Floating, 4500 V; declustering potential, −80 V; collision energy, −38.1 V; Ion Release Delay (IRD), 66; and Ion Release Width, 24. The quantirfication transition was m/z 635.2 for precursor ions and m/z 205.0721 for product ions. The quantification standard curves were prepared with external standards of each HMO, with concentrations of 0.15625, 0.3125, 0.625, 1.25, 2.5, 5, 10, and 20 pmol/μL and 1/x weighting.
Austin, S., et al. (2019). "Human Milk Oligosaccharides in the Milk of Mothers Delivering Term versus Preterm Infants." Nutrients 11(6).
Menzel, P., et al. (2021). "Concentrations of oligosaccharides in human milk and child growth." BMC Pediatr 21(1): 481.
Ren, X., et al. (2023). "Human Milk Oligosaccharides Are Associated with Lactation Stage and Lewis Phenotype in a Chinese Population." Nutrients 15(6).
Ferreira, A. L., et al. (2020). "Human Milk Oligosaccharide Profile Variation Throughout Postpartum in Healthy Women in a Brazilian Cohort." Nutrients 12(3).
Pell, L. G., et al. (2021). "The Human-Milk Oligosaccharide Profile of Lactating Women in Dhaka, Bangladesh." Curr Dev Nutr 5(12): nzab137.
